# NSCSO: a novel multi-objective non-dominated sorting chicken swarm optimization algorithm

**DOI:** 10.1038/s41598-024-54991-0

**Published:** 2024-02-21

**Authors:** Huajuan Huang, Baofeng Zheng, Xiuxi Wei, Yongquan Zhou, Yuedong Zhang

**Affiliations:** 1 College of Artificial Intelligence, Guangxi Minzu University, Nanning, 530006 China; 2 College of Electronic Information, Guangxi Minzu University, Nanning, 530006 China; 3 Guangxi Key Laboratory of Hybrid Computation and IC Design Analysis, Guangxi Minzu University, Nanning, 530006 China

**Keywords:** Multi-objective optimization, Meta-heuristic, Chicken swarm optimization algorithm, Fast non-dominated sorting, Multi-objective engineering design problems, Computational science, Computer science, Information technology, Scientific data

## Abstract

Addressing the challenge of efficiently solving multi-objective optimization problems (MOP) and attaining satisfactory optimal solutions has always posed a formidable task. In this paper, based on the chicken swarm optimization algorithm, proposes the non-dominated sorting chicken swarm optimization (NSCSO) algorithm. The proposed approach involves assigning ranks to individuals in the chicken swarm through fast non-dominance sorting and utilizing the crowding distance strategy to sort particles within the same rank. The MOP is tackled based on these two strategies, with the integration of an elite opposition-based learning strategy to facilitate the exploration of optimal solution directions by individual roosters. NSCSO and 6 other excellent algorithms were tested in 15 different benchmark functions for experiments. By comprehensive comparison of the test function results and Friedman test results, the results obtained by using the NSCSO algorithm to solve the MOP problem have better performance. Compares the NSCSO algorithm with other multi-objective optimization algorithms in six different engineering design problems. The results show that NSCSO not only performs well in multi-objective function tests, but also obtains realistic solutions in multi-objective engineering example problems.

## Introduction

Optimization challenges permeate various aspects of daily life, often manifesting as intricate multi-objective optimization problems (MOP). Effectively addressing these large-scale MOPs to attain satisfactory optimal solutions remains a formidable task. Unlike single-objective problems, MOPs defy evaluation through a singular criterion, demanding the comparison of multiple objectives. Complicating matters, these objectives frequently lack coordination and may even be mutually exclusive, making it impossible to simultaneously optimize all objectives optimally^1^. Consequently, the pursuit of a Pareto optimal solution emerges as the ultimate goal for MOPs^2^, aiming to strike a balance among diverse optimization objectives.

Historically, early attempts at MOP solutions employed the direct search method^3^. While this approach doesn’t directly tackle MOPs, it transforms the problem into several single-objective instances using various techniques, subsequently solving them sequentially. Despite its ability to yield relatively stable results, the direct search method is confined to convex MOPs. For nonconvex MOPs, it falls short in ensuring the acquisition of a uniformly distributed solution, let alone a superior Pareto optimal solution. Consequently, group search methods gained prominence, with many evolving from meta-heuristic algorithms^4^. These strategies represent a shift towards addressing the complexities of MOPs by leveraging collective search mechanisms, steering away from the limitations associated with traditional direct search approaches.

When the meta-heuristic algorithm solves complex optimization problems, it can accumulate experience after each iteration and finally arrive at a set of optimal solutions through continuous iteration. Meta-heuristics can be divided into various types, including biology-based, physics-based, mathematics-based, chemistry-based, music-based, sport-based, social-based, light-based, and water-based^5^. The sperm swarm optimization (SSO) is biological-based^6^; the archimedes optimization algorithm (AOA) is physics-based^7^; the league championship algorithm (LCA) is sport-based^8^; the harmony search (HS) is based on music^9^; the optics inspired optimization (OIO) is based on light^10^. Due to their low computational cost, these algorithms have been applied in real life, such as in sports^11^, medicine^12^, modeling^13^, etc. Most of these meta-heuristic algorithms and their application scenarios are single-objective problems. To address the MOP problem, it is necessary to extend the current set of meta-heuristic algorithms.

The multi-objective algorithm in the population search approach can randomly assign positions in the search space, perform autonomous learning and updating, and finally output a solution set with uniform distribution and small error value. Researchers can choose suitable solutions according to their own needs. Schaffer summarized the characteristics of prior, posterior, and interactive methods, tested the feasibility of these methods, and proposed the vector evaluation genetic algorithm (VEGA)^14^, which made pioneering work for the population search method. Goldberg proposed the non-dominated sorting and the Niche Technique to solve the MOP problem, which is of great significance to subsequent research^15^. A large number of multi-objective optimization algorithms have subsequently emerged, such as the multi-objective sperm fertilization procedure (MOSFP)^16^. The performance and stability of multi-objective optimization algorithms are constantly optimized, and such algorithms have been used in the direction of practical applications in industry, biology, economics et al. Among them, Ndao et al. applied multi-objective design optimization to electronic cooling technology and performed a comprehensive analysis and comparison^17^. For the high size of the internal permanent magnet synchronous motor (IPMSM) and the huge computational cost of finite element analysis^18^, Sun designed a new multi-objective optimization strategy that provides a solution with better performance and reduced computational cost^19^. Wind energy is a harmless and renewable clean energy source, and Liu et al. summarized many multi-objective optimization frameworks applied to wind energy prediction techniques^20^. In these studies, the multi-objective optimization algorithm provides researchers with better decision solutions, which is sufficient to show that this approach has many advantages in solving real-life MOP. Since Wolpert and Macready proposed the NFL theorem and proved that although a portion of MOP can be solved with the currently available technology^21^, there is still a portion of MOP that cannot be solved at the moment, and therefore new algorithms need to continue to be developed.

The chicken swarm optimization (CSO) algorithm, introduced by Meng et al.^22^, is a biologically-inspired meta-heuristic that mimics the hierarchical order, foraging, and learning behaviors observed in chickens. In the realm of solving single-objective problems, CSO has demonstrated notable strengths, including rapid convergence, high accuracy, and robustness. Despite these advantages, applying CSO directly to multi-objective problems (MOP) has proven challenging, underscoring the significance of exploring this research direction. Dinghui et al. conducted comprehensive testing of the CSO algorithm, employing techniques such as Markov chain analysis to establish its exceptional convergence performance^23^. This empirical validation ultimately confirmed the algorithm’s global convergence. Leveraging these findings, there is a compelling motivation to extend the applicability of CSO to MOP without deviating from its core principles. The objective is to ensure convergence while enhancing the algorithm’s capability to furnish optimal solutions aligned with true values for multi-objective scenarios.

In pursuit of this objective, the present study proposes the non-dominated sorting chicken swarm optimization (NSCSO) Algorithm. This extension builds upon the foundations of CSO while introducing modifications tailored to address the intricacies of solving multi-objective problems. The overarching goal is to broaden the scope of CSO applications, empowering the algorithm to deliver precise and reliable data in diverse multi-objective settings. Such advancements aim to facilitate decision-making processes for stakeholders by providing them with a repertoire of accurate solutions to choose from.

The main contributions of this paper are the following four points:

- Assign ranks to individuals in the chicken swarm using fast non-dominance sorting.
- In order to sort different particles in the same rank, the concept of crowding distance is introduced.
- Use the elite opposition-based learning strategy to make it easier for individual roosters to explore the direction of the optimal solution.
- It evaluated the performance of NSCSO with fifteen multi-objective benchmark functions and six engineering design strengths.

The main framework of this paper is described next. In “Literature review” section, the basic definition of multi-objective optimization and the current state of research are described. “Chicken swarm optimization algorithm” section introduces the concept of the basic CSO algorithm in terms of the main ideas and so on. An introduction to the NSCSO algorithm is placed in “The multi-objective non-dominated sorting chicken swarm optimization algorithm” section. “Experimental results and analysis” section then discusses the algorithm with experiments and results. In order to better illustrate the advantages of this algorithm in solving practical problems, in “Engineering design problems” section, the NSCSO algorithm is used to solve six engineering cases. Finally, “Conclusions” section summarizes our work and provides plans and suggestions for future work.

## Literature review

### Multi-objective optimization

Generally, the number of objective functions is two or more, and a problem with multiple decision variables is called a multi-objective optimization problem (MOP). The definition that is widely adopted in this domain is as follows^24^:

1 $$ Minimize:\;y = F(x) = (f_{1} (x),f_{2} (x), \ldots ,f_{m} (x)), $$

2 $$ {\text{Subject to}}: g_{i} (x) \le 0,i = 1,2, \ldots ,q, $$

3 $$ h_{j} (x) = 0,j = 1,2, \ldots ,p, $$

4 $$ x = (x_{1} ,x_{2} , \ldots ,x_{n} ) \in X \subset R^{n} , $$

5 $$ y = (y_{1} ,y_{2} , \ldots y_{m} ) \in Y \subset R^{m} , $$

where $m$, $n$ correspond to the number of objective functions and decision variables, respectively; is called the decision vector, $x_{i} (i = 1,2, \ldots ,n)$ is the decision variable, the decision space is $X$, with n dimensions; $y$ is the objective vector, $Y$ is the n-dimensional objective space; $q$, $p$ are the number of inequality constraints and equation constraints, respectively; $g_{i} (x)$ is the *i*th in-equality $x$ constraint; $h_{j} (x)$ is the *j*th equality constraint.

It can be known from the above formula that there are multiple different objective functions in MOP. In most cases of MOP, the interests of each objective may affect each other, and the improvement of one party may cause performance degradation of other parties. Therefore, for MOP, the Pareto optimal solution set is the optimal solution that is ultimately desired, and this set contains many solutions, even an infinite number of solutions. Therefore, it is necessary to choose the part of Pareto optimal solutions to use according to our actual needs^25^. The following will define the concepts such as Pareto:

#### Definition 1

(*Pareto Dominance*) Suppose $x_{1} ,x_{2} \in X_{f}$, $x_{1}$ Pareto dominance $x_{2}$(denoted as $x_{1} \prec x_{2}$), if and only if the Eq. (6) holds:

6 $$ \begin{gathered} \forall i = 1,2, \cdots ,m:f_{i} (x_{1} ) \le f_{i} (x_{2} ) \wedge \hfill \\ \exists i = 1,2, \cdots ,m:f_{i} (x_{1} ) < f_{i} (x_{2} ), \hfill \\ \end{gathered} $$

where $x_{1}$ is better than $x_{2}$ when at least one of the fitness values $f_{i} (x_{1} )$ of $x_{1}$ is better than the fitness value $f_{i} (x_{2} )$ of $x_{2}$. This is called Pareto dominance, and is denoted using $x_{1} \prec x_{2}$.

#### Definition 2

(*Pareto Optimality*) The specific conditions for satisfying the Pareto optimal solution are as follows:

7 $$ \neg \exists x \in X_{f} :x \prec x^{*} , $$

where the Pareto optimal solution also becomes a non-inferior solution or an efficient solution. In the decision space $X_{f}$, if the number of times the decision vector $x$ is dominated by other decision vectors is 0, it is the Pareto optimal solution.

#### Definition 3

(*Pareto Optimality Set*) The Pareto optimal solution set, which contains all Pareto optimal solutions obtained by Definition 2. Is called the Pareto set (PS), where:

8 $$ PS = \{ x^{*} \} = \{ x \in X_{f} |\neg \exists x^{\prime} \in X_{f} :x^{\prime} \prec x^{*} \} . $$

#### Definition 4

(*Pareto Optimality Front*) Pareto Frontier (PF). The result obtained by projecting the Pareto optimal solution into the target search space is PF, namely:

9 $$ PF = \{ F(x)|x \in PS\} . $$

### Related work

The main method to solve MOP is to use the multi-objective optimization algorithm (MOA), which can automatically search for the optimal value in the target space through multiple iterations and determine the direction of the next movement through experience, and is a powerful tool to solve MOP. The multi-objective evolutionary algorithm (MOEA) and the multi-objective swarm intelligence algorithm belong to MOA^26^.

MOEA includes many kinds, the multi-objective genetic algorithm (MOGA) was proposed by Murata and Ishibuchi^27^. In the selection process, the algorithm randomly assigns multiple objective function weights, and the elite individuals are selected from the Pareto optimal solution, and then passed to the next generation. Non-dominated sorting based genetic algorithm (NSGA)^28^, was proposed by Srinivas and Kalyanmoy, NSGA uses the genetic algorithm and non-dominated sorting strategy to find Pareto optimal solution, but it requires a lot of computational costs to solve MOP. Then Deb et al. improved NSGA and proposed the NSGA-II algorithm^29^. With the continuous research on MOEA, the concept of external archives was proposed. This method can retain the obtained non-dominated solutions. Through continuous iteration, solutions with good performance are added to the archives, and solutions with poor performance are deleted from the archives. To further enhance the ability of particles in MOEA to learn excellent individuals and improve the performance of the algorithm, an elite strategy is studied, which ensures that MOEA learns the global optimal solution better. Zitzler and Thiele proposed the strength Pareto evolutionary algorithm (SPEA)^30^, which uses external archives to retain all obtained non-dominated solutions to evaluate individual fitness according to Pareto dominance relations. Subsequently, Zitzler et al. improved SPEA and proposed the SPEA2 algorithm^31^. Combining the nearest neighbor density estimation technology and a new external archive filing strategy, it ensures that the boundary solution is not lost and improves the precision of the algorithm. The decomposition-based multi-objective evolutionary algorithm (MOEA/D) was proposed by Zhang and Li^32^, which is capable of converting MOP into scalar subproblems and performing simultaneous optimization of these subproblems, thus reducing the computational complexity.

Since most of the problems in MOP are NP-hard problems, while swarm intelligent optimization algorithms have great advantages for solving NP-hard problems, Therefore, many scholars began to study the use of multi-objective swarm intelligence optimization algorithm to solve MOP, among which the most classical algorithms include multi-objective particle swarm optimization algorithm (MOPSO)^33^, multi-objective simulated annealing algorithm (MOSA)^34^, multi-objective ant colony optimization algorithm (MOACO)^35^, and so on. Meanwhile, based on these original algorithms, other swarm intelligence optimization algorithms have been developed by researchers with their corresponding multi-objective versions. Mirjalili et al. proposed the multi-objective ant-lion optimization algorithm (MOALO)^36^, which maintains the main search mechanism of the basic ant-lion optimization algorithm. The Pareto optimal solution obtained so far is stored through an external archive. The individual ant lions are selected using the roulette strategy, and the selected ant lion guides the ants in their exploration. Most of the ideas of multi-objective population intelligence optimization algorithms retain the characteristics of single-objective algorithms, and obtain the Pareto optimal solution through external files or non-dominated sorting.

There are also many versions of algorithms developed to solve large-scale MOP problems. Liu et al. clustered decision variables into two categories and then used dimensionality reduction methods to represent variables that affect evolutionary convergence in low dimensions. They proposed an evolutionary algorithm for large-scale multi-objective decision problems based on clustering and dimensionality reduction, which achieved good performance^37^. Cao et al. proposed and discussed multi-objective large-scale distributed parallel particle swarm optimization algorithms for these multi-objective large-scale optimization problems, and looked forward to future research directions^38^. Li et al. used a fast cross-correlation identification algorithm to divide decision variables into different groups and then used a new coevolutionary algorithm to solve multi-objective optimization problems. Experimental results on large-scale problems showed that the algorithm was effective^39^. Allah et al. proposed a multi-objective orthogonal opposition-based crow search algorithm (M2O-CSA), and simulation results confirmed the effectiveness of the proposed M2O-CSA algorithm^40^.

After the description above, MOA has been developed a lot now, and some algorithms have shown better performance in real-life examples^41^. However, the NFL law shows that the field still needs to develop new algorithms for problem-solving, and although there are already many ways to solve MOP, there are still some MOP that no method can solve yet.

## Chicken swarm optimization algorithm

### Biological paradigm

Chicken swarm optimization (CSO) Algorithm was proposed by Meng et al.^42^. Usually, in a chicken swarm, there are several categories of roosters, hens, and chicks, and each chicken has its own corresponding identity, according to which it forages and learns from its own two parents.

The CSO algorithm is mainly designed by observing the hierarchical order, foraging behavior, and learning behavior of chickens as the core of the model design and location update design. Among them, the most important ideas of the CSO algorithm are as follows.

#### Defining order

Set each individual as a chicken in the flock, each individual has its own corresponding role, namely rooster, hen, and chick.

Each subgroup is led by one and only one rooster, who has the highest status and is the best adopted of the subgroup. Chicks are the vulnerable group in the population, so their fitness value is the worst. The remaining individuals are hens. Mother chicks are selected by random selection among the hens and assigned chicks to them.

After each G iteration, the hierarchy of each individual in the swarm will be reset according to its fitness, and the dominance and mother–child relationships will also be updated.

#### Foraging order

The rooster has the highest status in the subgroup and will lead his subgroup in foraging, while the hen follows the rooster in the subgroup in foraging or goes to plunder the food of other chickens, provided that the target food is good for itself. Chicks are the weakest of the breed and can only follow their mothers in foraging.

### Mathematical models

It is defined that the whole chicken swarm consists of $N$ individuals, then the number of roosters, hens, mothers, and chicks can be denoted as $N_{R} ,N_{H} ,N_{M} ,N_{C}$. Then $x_{i,j}^{t}$ denotes the position of the *i*th chicken in the *j*th dimension of the *t*th iteration in the D-dimensional space, and M represents the maximum number of iterations $(i \in (1,2, \ldots ,N),j \in (1,2, \ldots ,D),t \in (1,2, \ldots ,M))$.

The rooster is the individual with the best fitness in the subgroup, and it can decide the foraging direction by itself. According to the above expression method, Then the update formula of this part is shown in Eqs. (10) and (11):

10 $$ x_{i,j}^{t + 1} = x_{i,j}^{t} \times [1 + N(0,\sigma^{2} )], $$

11 $$ \sigma^{2} = \left\{ {\begin{array}{*{20}c} {1,} & {f_{i} \le f_{k} } \\ {\exp (\frac{{f_{k} - f_{i} }}{{|f_{i} | - \varepsilon }}),} & {f_{i} > f_{k} } \\ {k \in [1,N_{R} ],} & {k \ne 1} \\ \end{array} } \right., $$

where, $N(0,\sigma^{2} )$ is the normal distribution, 0 is the mean, and $\sigma^{2}$ is the variance; $k$ is the index of another rooster randomly selected in the population, the fitness of rooster $i$ and rooster $k$ are denoted by $f_{i}$ and $f_{k}$; $\varepsilon$ is the smallest number in the computer, and its role is to prevent errors when the denominator is 0.

The hens can only follow the roosters in the subgroup to forage, and the hens can also rob other individuals of better-quality food than themselves. The position update formula of the hen is shown in Eqs. (12)–(14).

12 $$ x_{i,j}^{t + 1} = x_{i,j}^{t} + S_{1} R_{1} (x_{{r_{1} ,j}}^{t} - x_{i,j}^{t} ) + S_{2} R_{2} (x_{{r_{2} ,j}}^{t} - x_{i,j}^{t} ), $$

13 $$ S_{1} = \exp \left( {\frac{{f_{i} - f_{{r_{1} }} }}{{abs(f_{i} ) + \varepsilon }}} \right), $$

14 $$ S_{2} = \exp (f_{{r_{2} }} - f_{i} ), $$

where $R_{1}$ and $R_{2}$ satisfy the condition of $R_{1} ,R_{2} \in \left[ {0,1} \right]$ and are two random numbers; $r_{1}$ is a rooster in the hen’s subgroup; $r_{2}$ is a randomly selected chicken, which can be either a rooster or a hen, but its fitness is better than hen $i$, while it can’t be chicken $r_{1}$ and $i$, $\varepsilon$ is the smallest number in the computer.

Chicks can only move with their mother chickens, and they are a vulnerable group in the population. The position update formula of the chick is shown in Eq. (15).

15 $$ x_{i,j}^{t + 1} = x_{i,j}^{t} + FL(x_{m,j}^{t} - x_{i,j}^{t} ). $$

Among them, $m$ is the chicken mother of a chick $i$, and $FL$ represents its adjustment parameter to follow the chicken mother, which is usually a random number between $[0,2][0,2]$. Algorithm 1 is pseudocode for the standard CSO algorithm.

**Algorithm 1:**
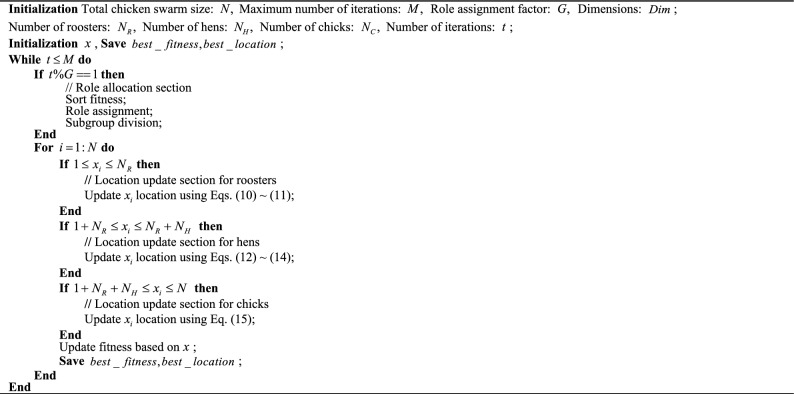
CSO algorithm.

## The multi-objective non-dominated sorting chicken swarm optimization algorithm

Researched and developed the non-dominated sorting chicken swarm optimization (NSCSO) algorithm. On the premise of not changing the chicken swarm optimization (CSO) algorithm framework introduced in “Chicken swarm optimization algorithm” section, the NSCSO algorithm adds a fast non-dominated sorting strategy and a crowding degree strategy. There are two purposes for utilizing these two strategies: Firstly, to be able to find non-dominated solutions by dividing all particles into non-dominated ranks. The second is because the different hierarchies in CSO are established by differentiating individual fitness. In a multi-objective optimization problem (MOP), the goodness of a solution cannot be judged by the fitness of an objective alone, so the individual is ranked by the non-dominated sequence and the crowding degree together, to determine which group the individual belongs to.

**Algorithm 2:**
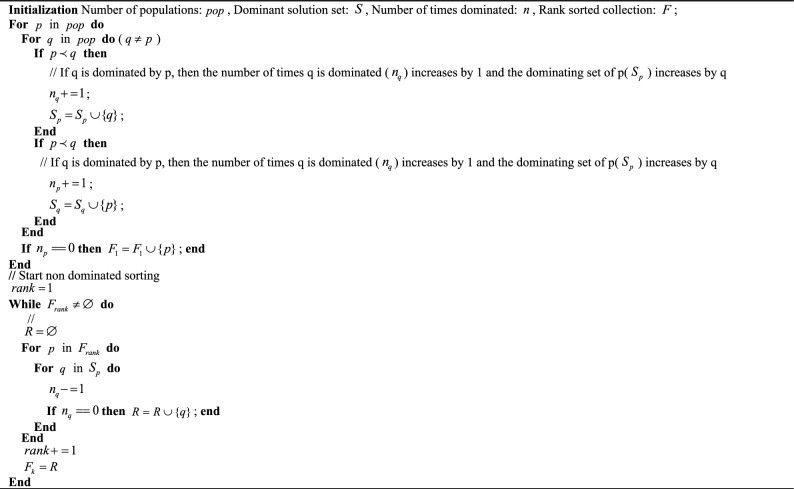
Fast non-dominated ranking.

### Fast non-dominated sorting

Fast non-dominant sorting sets two parameters for all search particles, the number of dominations $n_{i}$ and the dominating set $S_{i}$. The main operation process is described below:

Step 1: Calculate $n_{i}$ and $S_{i}$ of all particles. For example, if the particles $i$, $j$ satisfy $i \prec j$, the $n_{i}$ particle $i$ is incremented by 1, and the index of the particle $i$ is put into the $S_{j}$ set of the particle $j$.
Step 2: Put the particles with $n_{i} = 0$ into $F_{1}$, the Pareto optimal solution set is $F_{1}$ because of $n_{i} = 0$.
Step 3: Visit the $S_{i}$ of all particles in $F_{1}$, and decrement the $n_{i}$ of its members by one.
Step 4: Put the particles with $n_{i} = 0$ into the corresponding $F_{rank}$ at this time, and visit the dominating set $S_{i}$ in the corresponding $F_{rank}$, decrement the $n_{i}$ of the members by 1, and repeat *Step 4* until $F_{rank}$ is empty.

**Figure 1:**
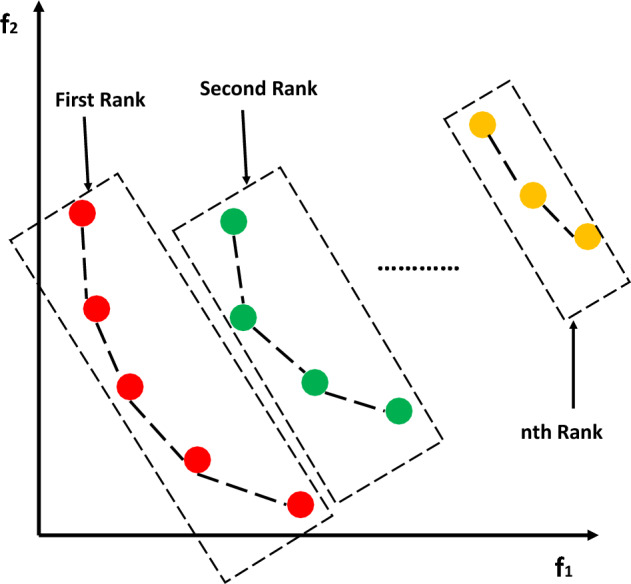
Correspondence of $$F_{rank}$$**.**

Figure 1 shows the correspondence of $F_{rank}$, where First Rank is the Pareto optimal solution, and the particles in this Rank are dominated is 0; The particles in Second Rank are dominated by at least one particle in First Rank. Other ranks are analogous. Algorithm 2 shows the pseudocode for fast non-dominated sorting.

### Crowding distance strategy

**Figure 2:**
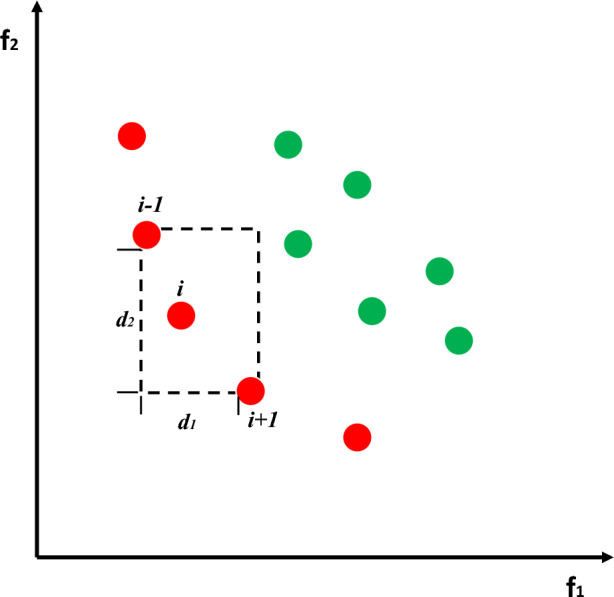
The crowding distance strategy.

According to the above-mentioned fast non-dominated sorting method, the particles can be accurately classified into multiple ranks with different levels. Among them, $F_{1}$ is the Pareto optimal solution, and the particle quality in this rank is the best. With the increase of $rank$ in $F_{rank}$, the quality of the particles in the corresponding rank becomes worse. Although fast non-dominated sorting can be used to sort particles according to their mass, there is a high probability that multiple particles will appear at the same level of $F_{rank}$. The quality of these particles can no longer be distinguished, so the NSCSO cannot smoothly assign the role to each particle. This problem is solved with the introduction of the crowding degree strategy. The strategy sets a predefined distance for the particles in different Ranks, calculates the distance of each nearest particle within the preset distance, and then performs a normalization process as its crowding degree. In this way, particles of the same rank can be further sorted according to the degree of congestion. The calculation formula of crowding degree is shown in Eq. (16).

16 $$ D^{i} = \sum\limits_{j = 1}^{njob} {\frac{{abs(f_{j}^{i + 1} - f_{j}^{i - 1} )}}{{abs(f_{j}^{\max } - f_{j}^{\min } )}}} , $$

where $D^{i}$ denotes the crowding degree of the *i*th particle in a certain $F_{rank}$; $njob$ is the number of objective functions of the problem; $f_{j}^{i + 1}$ and $f_{j}^{i - 1}$ are the fitness values of the (*i* + 1)th and the (*i − *1)th particle in the *j*th objective function, respectively; $f_{j}^{\max }$ and $f_{j}^{\min }$ are the maximum and minimum values of the *j*th objective function, respectively. The crowding distance strategy in Fig. 2, $d_{1}$ is the length of the dotted quadrilateral of particle $i - 1$ and particle $i + 1$, $d_{1}$ is the width 2 of the dotted quadrilateral of particle $i - 1$ and particle $i + 1$, and the sum of the length and width is the distance between particle $i$ and its two adjacent individuals in each sub-unit. The sum of distance differences on the objective function.

### Particle movement

The NSCSO follows the update method of the single-objective CSO algorithm. In order to enable particles to perform multiple iterative searches in the multi-target search space, the update method of different populations has been modified.

First, in the rooster update of the original CSO algorithm, it is necessary to compare the fitness of the two roosters. If rooster $k$ is better than rooster $i$, the rooster $i$ will move to the position of the rooster $k$; otherwise, the rooster $i$ will continue to explore other spaces. In the MOP, quality of the particles cannot be judged by the particle fitness alone, it is necessary to modify this part to make it suitable for searching in multi-object space.

The main idea is as follows: First compare the $F_{rank}$ levels of rooster $i$ and rooster $k$, if rooster $i$’s rank is higher than rooster $k$ ($F_{rank}$’s rank is higher than $F_{rank + 1}$’s rank), the rooster $i$ will continue to explore other spaces; If the rank of the rooster $i$ is lower than that of the rooster $k$, then the direction of movement of rooster $i$ will point to the position of rooster $k$; If the rank of the rooster $i$ and rooster $k$ are the same, then compare the crowding degree. If the crowding degree of the rooster $i$ in the same rank is higher than that of the rooster $k$, then the rooster $i$ will continue to explore other spaces; if the crowding degree of the rooster $i$ in the same rank is lower than that of the rooster $k$, then the rooster $i$ will move to the position of the rooster $k$. As mentioned above, NSCSO sorts the particles of the entire population by fast non-dominated ranking and crowding degree strategy. The indexes of the particles are sorted by the quality of the particles from good to bad, so the quality of the rooster $i$ and the rooster $k$ can be compared directly through the index of each chicken, the better the mass of the particle the smaller the index.

In the original CSO algorithm, the value of $\sigma^{2}$ is affected by the fitness of rooster $i$ and rooster $k$. In the multi-objective problem, there are multiple fitness values. Without changing the fundamental principle, the calculation of $\sigma^{2}$ is performed by taking the mean value of the fitness of each objective function. The revised update method is shown in Eqs. (17) and (18).

17 $$ x_{i,j}^{t + 1} = x_{i,j}^{t} \times [1 + N(0,\sigma^{2} )], $$

18 $$ \sigma^{2} = \left\{ {\begin{array}{*{20}c} {1,} & {index_{i} \le index_{k} } \\ {\frac{{\sum\limits_{n = 1}^{njob} {\exp \left( {\frac{{f_{k,n} - f_{i,n} }}{{|f_{i,n} | - \varepsilon }}} \right)} ,}}{njob}} & {index_{i} > index_{k} } \\ {k \in [1,N_{R} ],} & {k \ne 1} \\ \end{array} } \right., $$

where $f_{k,n}$ and $f_{i,n}$ represents the fitness function values of rooster $k$ and rooster $i$ in the *n*th objective function. $index_{i}$ and $index_{k}$ is the index of rooster $i$ and rooster $k$; $njob$ is the number of total objective functions of the problem.

In the original CSO algorithm, there are two important parameters in the hen population update method, $S_{1}$ and $S_{2}$. They represent two types of hen behaviors: $S_{1}$ simulates the foraging and learning behavior of the hen following the roosters in her population; $S_{2}$ simulates the competition between the hen and other chickens. These two parameters are also calculated utilizing fitness values, and to adapt them to the multi-objective problem, NSCSO also takes a mean value approach to their calculation, as shown in Eqs. (19)–(21).

19 $$ x_{i,j}^{t + 1} = x_{i,j}^{t} + S_{1} R_{1} (x_{{r_{1} ,j}}^{t} - x_{i,j}^{t} ) + S_{2} R_{2} (x_{{r_{2} ,j}}^{t} - x_{i,j}^{t} ), $$

20 $$ S_{1} = \frac{{\sum\limits_{n}^{njob} {\exp \left( {\frac{{f_{i,n} - f_{{r_{1} ,n}} }}{{abs(f_{i,n} ) + \varepsilon }}} \right)} }}{njob}, $$

21 $$ S_{2} = \frac{{\sum\limits_{n}^{njob} {\exp (f_{{r_{2} ,n}} - f_{i,n} )} }}{njob}, $$

where $njob$ is the number of total objective functions of the problem; $f_{i,n}$, $f_{{r_{1} ,n}}$ and $f_{{r_{2} ,n}}$ represent the fitness function values of hen $i$, rooster $r_{1}$, and chicken $r_{2}$ in the *n*th objective function.

### Elite opposition-based learning strategy

The main idea of the opposition-based learning (OBL) is to map the current particle to its opposite position, this strategy was proposed by Tizhoosh. Since the CSO algorithm has the rooster population as the supreme leader^43^, leading the entire chicken swarm in the search of space. If the rooster falls into a local optimum, the convergence accuracy and speed deteriorate as the other individuals can only learn from the rooster, resulting in all particles approaching the local optimum. The introduction of the elite OBL strategy can provide a variable to assist the rooster group to move in the opposite direction when the rooster group cannot jump out of the local optimum for a long time, thereby guiding other individuals to learn from it and improving individual quality. The specific implementation of this strategy is shown in Eq. (22).

22 $$ x_{i}^{*} = lb + ub - x_{i} , $$

where $x_{i}^{{}}$ is the current position of the rooster $i$; $x_{i}^{*}$ is the position obtained by the rooster $i$ elite OBL; $lb$ and $ub$ are the upper and lower limits of the particle. The NSCSO performs elite OBL on the rooster population after every $G$ iterations. And set a random learning probability $p$ ($p \in [0,1]$), each rooster generates a random number $p^{*}$ ($p^{*} \in [0,1]$), when $p^{*} < p$, perform elite OBL, otherwise do not learn. Finally, if $x_{i}^{*} \prec x_{i}$, then replace $x_{i}^{*}$ with $x_{i}^{{}}$ and add it to subsequent iterations, otherwise, $x_{i}^{{}}$ will still be used. The pseudocode of the Elite OBL Strategy is shown in Algorithm 3.

Control experiments for the NSCSO algorithm using the elite OBL strategy and the algorithm not using this strategy will be given in “Experimental results and analysis” section.

**Algorithm 3:**
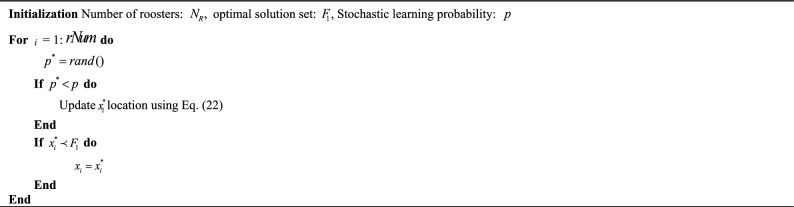
Elite opposition-based learning strategy.

### NSCSO algorithm steps

**Figure 3:**
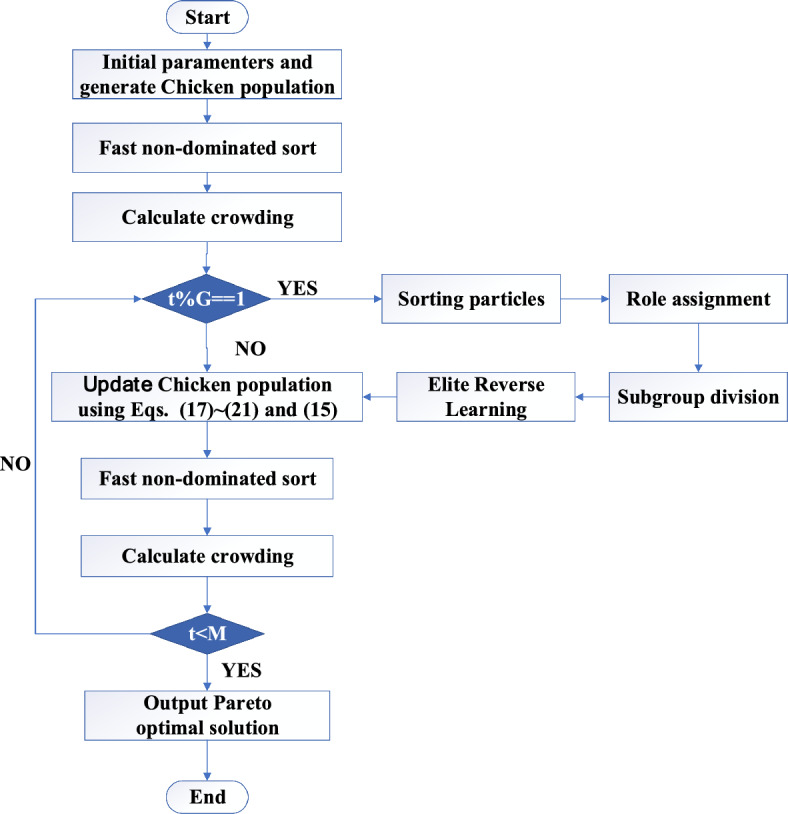
The flowchart of the proposed NSCSO algorithm.

Figure 3 is a flow chart of the NSCSO algorithm, and Algorithm 4 is the pseudocode of the NSCSO.

The specific steps of each iteration of the NSCSO algorithm are as follows:

Step 1: Initialization: dimension $Dim$; population size $N$; each population size $N_{R} ,N_{H} ,N_{m} ,N_{C}$; order redefinition parameter $G$; the number of iterations $t$; maximum number of iterations $M$.
Step 2: Randomly generate individual positions and calculate individual fitness. Perform fast non-dominated sorting and calculate crowding degree.
Step 3: If $t\% G = = 1$, first perform order allocation and allocate all individuals into roosters, hens, and chicks according to the sorting results. Roosters are assigned their dominant hens and chicks, and chicks are assigned mother chickens; elite OBL strategy is then performed on the roosters. Otherwise go to Step 4.
Step 4: Each updates its position according to its role and calculates its fitness value.
Step 5: Performs non-dominated sorting on the newly arrived individuals, calculates their crowding degree, and updates the individuals.
Step 6: If $t < = M$, then return to Step 3; if $t > M$, output Pareto optimal solution.

**Algorithm 4:**
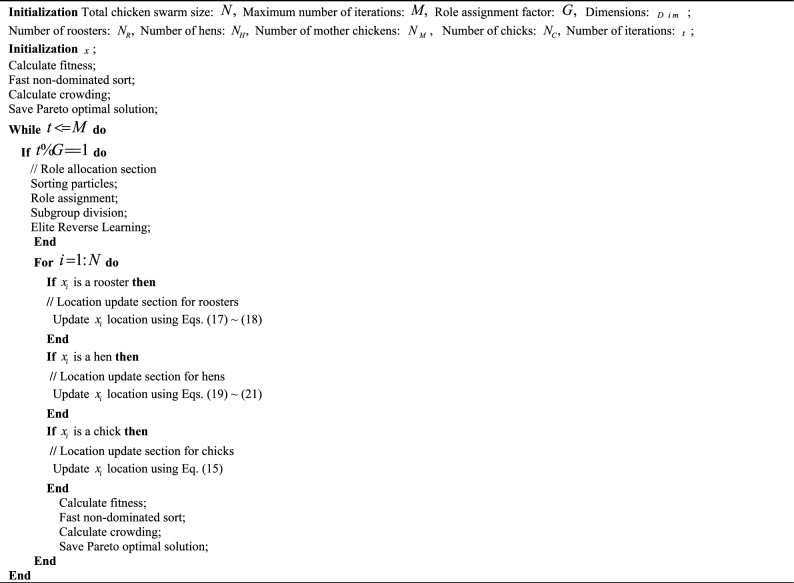
NSCSO algorithm.

### The computation complexity of NSCSO

In terms of time complexity, the time complexity of the initialization phase of NSCSO is $O(N \times njob)$, the time complexity of fast non-dominated sorting is $O(M \times njob \times N^{2} )$, the time complexity of calculating crowding degree is $O(M \times njob \times N \times \log N)$, and the time complexity of redefining order is $O(M/G \times N)$. The time complexity required to update the particle position is $O(M \times Dim \times N)$, and the time complexity required to calculate the objective function is $O(M \times N \times {\text{cos}}t(fobj))$, so the total time complexity of the NSCSO algorithm is $O(N \times njob + M \times N \times (njob \times N + njob \times \log N + 1/G + Dim + {\text{Cos}} t(fobj))),$ where $N$ is the population size, $Dim$ is the population dimension, $njob$ is the number of objective functions, $M$ is the maximum number of iterations, $G$ is the order redefinition parameter, and ${\text{cos}}t(fobj)$ is the cost of the objective function.

In terms of space complexity, the NSCSO algorithm needs to consider the space complexity of the population initialization, that is, the space complexity is $O(N \times njob)$.

## Experimental results and analysis

In this section, the NSCSO algorithm and six other algorithms are tested using 15 different benchmarking functions, and four performance metrics are utilized as evaluation criteria and references, and the results are fully discussed at the end.

### Experimental environment

The experiments of the proposed NSCSO algorithm were tested in MATLAB R2019b under 64-bit Windows 10 with a hardware configuration of Intel Core i5-8300H 2.30 GHz processor and 8 GB RAM.

### Benchmark function test

For the proposed NSCSO algorithm, test experiments were conducted on 15 different benchmark functions. Five test functions were selected from the ZDT^44^, DTLZ^45^, and WFG^44^ test sets respectively for algorithm performance testing. Their ZDT1–ZDT4 and ZDT6 are dual-objective tests, and DTLZ2, DTLZ4–DTLZ7, and WFG4–WFG8 are triple-objective tests.

### Algorithm parameters

This paper compares NSCSO with other six excellent multi-objective optimization algorithms, which are: LMEA^46^, WOF^47^, multi-objective slime mould algorithm (MOSMA)^48^, DGEA^49^, multi-objective artificial hummingbird algorithm (MOAHA)^50^ and multi-objective stochastic paint optimizer (MOSPO)^51^. In test experiments, these algorithms were run independently on the benchmark function 30 times with 1000 iterations per iteration. Each algorithm has a population size of 100, the ZDT and DTLZ test set dimensions of 10, and the WFG test set dimensions of 12. The parameter settings of all algorithms are shown in Table 1.

**Table 1:** Parameters of algorithms.

Algorithm	Parameters
LMEA^46^	nSel: 5 nPer: 50 nCor: 5
WOF^47^	Gamma: 4, Groups: 2 Psi: 3 T1: 1000, T2: 500 Delta: 0.5
MOSMA^48^	Number of iterations: $t$, The maximum number of iterations: $T$, $a = \arctan h(1 - (t/T))$ $b = 1 - (t/T)$ Flow speed control parameters: $v_{b} = [ - a,a],v_{c} = [ - b,b]$
DGEA^49^	Refno: 10
MOAHA^50^	Flight factor: ${\text{r}} = [0,1]$ Boot factor: $a = N(0,1)$ Territorial factor: $b = N(0,1)$
MOSPO^51^	Grids number per each dimension: $nGrid = 10$ Grid inflation: $\alpha = 0.1$ Archive member selection pressure: $\gamma = 2$ Leader selection pressure parameter: $\beta = 4$
NSCSO	Order redefinition parameter: $G = 10$ population size: $N = 100$ Number of roosts: $N_{R} = 0.15 \times N$ Number of hens: $N_{H} = 0.7*N$ Number of chicks: $N_{C} = 0.15*N$

### Performance metrics

In this paper, the algorithm is tested from multiple angles using different performance metrics. The specific usage of these performance indicators is as follows.

Generational distance (GD) has a simple design and good practicability and is suitable for comparison between multiple algorithms^52^. GD indicates the distance between the Pareto optimal solution derived by the algorithm and the true value, and its formula is shown in Eq. (23).

23 $$ GD = \frac{{\sqrt {\sum\limits_{i = 1}^{N} {d_{i}^{2} } } }}{N}, $$

where $d_{i}$ denotes the Euclidean distance between the *i*th solution in the target space and the nearest solution in the true value; $N$ is the solution obtained by the algorithm.

Inverse generational distance (IGD) tests the comprehensive performance of the algorithm^52^. IGD uses the average distance from the solution point in the true value to the solution point found by the algorithm. The smaller the IGD, the better the convergence and diversity of the solution obtained by the algorithm. IGD is the inverse mapping of GD. The calculation formula is shown in Eq. (24).

24 $$ IGD = \frac{{\sqrt {\sum\limits_{i = 1}^{PT} {d_{i}^{2} } } }}{PT}, $$

where $PT$ is the true value of Pareto, and $d_{i}$ represents the Euclidean distance between $PT$ and the nearest solution point of $N$.

Spatial metrics (SP) can be applied to multi-objective optimization problems with more than two dimensions^53^. SP evaluates the uniformity of the distribution of the solution obtained by the algorithm in the target space. The smaller the SP, the more uniform the distribution of the solution. Its calculation formula is shown in Eqs. (25) and (26).

25 $$ SP = \sqrt {\frac{1}{n - 1}\sum\limits_{i = 1}^{n} {(\overline{d} - d_{i} )^{2} } } , $$

26 $$ d_{i} = \min \sqrt {\sum\limits_{m = 1}^{njob} {|f_{m}^{i} - f_{m}^{j} |} } , $$

where $n$ is the number of solutions obtained by the algorithm; $njob$ is the number of objective functions; $d_{i}$ denotes the Euclidean distance between the *i*th solution and its nearest solution point; $\overline{d}$ is the average of $d_{i}$.

Maximum spread (MS), MS is used to measure the degree of coverage of the resulting solution to the true value^54^. It is calculated as shown in Eq. (27).

27 $$ MS = \sqrt {\sum\limits_{i = 1}^{njob} {\max (d(a_{i} ,b_{i} ))} } , $$

where $njob$ is the number of objective functions; $d(a_{i} ,b_{i} )$ denotes the Euclidean distance between the maximum value $a_{i}$ and the minimum value $b_{i}$ of the resulting solution in the *i*th objective.

### Performance evaluation

Table 2 presents the performance metrics of the NSCSO algorithm using the reverse elite learning strategy and the algorithm without this strategy in ZDT1. Where NSCSO-noEOBL represents an algorithm that does not use this strategy. It can be seen that after using this strategy, the stability and accuracy of the NSCSO algorithm have been improved, which proves the feasibility and effectiveness of introducing this strategy.

**Table 2:** Strategy controlled trials on ZDT1.

Method		GD	IGD	SP	MS
NSCSO	Mean	**6.03 × 10**^**–05**^	**4.93 × 10**^**–03**^	**6.95 × 10**^**–03**^	**3.97 × 10**^**–01**^
	**Std**	**3.48 × 10**^**–05**^	**2.58 × 10**^**–04**^	**4.98 × 10**^**–04**^	**3.87 × 10**^**–02**^
NSCSO-noEOBL	Mean	1.31 × 10^–06^	6.926 × 10^–04^	8.95 × 10^–03^	7.97 × 10^–01^
	Std	5.86 × 10^–05^	8.45 × 10^–04^	5.50 × 10^–04^	4.45 × 10^–02^

Significant values are in bold.

Tables 3, 4, 5 and 6 respectively count the mean and standard deviation of the results obtained by running 30 times, 1000 times each iteration, in different test functions for seven algorithms. Figures 4, 5, 6, 7, 8, 9, 10, 11, 12, 13, 14, 15, 16 and 17 show the Pareto frontier (PF) distributions obtained by algorithms such as NSCSO in the ZDT6 and DTLZ5 test problems. Figures 18, 19, 20, 21, 22, 23, 24, 25, 26 and 27 show the distribution of real PF and PF obtained by NSCSO for different test functions.

**Table 3:** The GD values obtained by all algorithms.

Problem		LMEA	WOF	MOSMA	DGEA	MOAHA	MOSPO	NSCSO
Bi-objective test function								
ZDT1	Mean	6.63 × 10^–05^	**4.51 × 10**^**–05**^	2.48 × 10^–04^	4.93 × 10^–05^	2.07 × 10^–04^	6.38 × 10^–04^	6.03 × 10^–05^
	Std	4.35 × 10^–05^	4.48 × 10^–05^	3.53 × 10^–05^	1.74 × 10^–04^	3.57 × 10^–05^	2.29 × 10^–04^	**3.48 × 10**^**–05**^
ZDT2	Mean	9.47 × 10^–05^	9.45 × 10^–05^	3.12 × 10^–04^	9.45 × 10^–05^	1.43 × 10^–04^	5.14 × 10^–04^	**9.29 × 10**^**–05**^
	Std	6.25 × 10^–06^	5.70 × 10^–06^	2.94 × 10^–05^	5.17 × 10^–06^	2.18 × 10^–05^	2.35 × 10^–04^	**4.72 × 10**^**–06**^
ZDT3	Mean	1.98 × 10^–04^	2.12 × 10^–04^	2.21 × 10^–04^	5.78 × 10^–03^	1.93 × 10^–04^	4.26 × 10^–04^	**1.77 × 10**^**–04**^
	Std	1.38 × 10^–05^	1.67 × 10^–04^	**1.26 × 10**^**–05**^	2.56 × 10^–05^	1.51 × 10^–05^	1.19 × 10^–04^	1.44 × 10^–05^
ZDT4	Mean	1.68 × 10^–04^	–	1.81 × 10^–04^	5.14 × 10^–05^	1.63 × 10^–04^	–	**5.00 × 10**^**–05**^
	Std	3.57 × 10^–04^	–	**3.61 × 10**^**–05**^	1.83 × 10^–04^	4.93 × 10^–05^	–	5.32 × 10^–05^
ZDT6	Mean	1.15 × 10^–03^	1.22 × 10^–03^	6.02 × 10^–02^	**1.11 × 10**^**–03**^	3.72 × 10^–02^	9.29 × 10^–02^	3.18 × 10^–03^
	Std	1.86 × 10^–03^	5.18 × 10^–04^	1.61 × 10^–02^	**4.24 × 10**^**–04**^	3.39 × 10^–02^	9.21 × 10^–02^	5.73 × 10^–04^
Three-objective test function								
DTLZ2	Mean	6.78 × 10^–04^	7.77 × 10^–04^	1.94 × 10^–01^	7.64 × 10^–04^	1.94 × 10^–01^	1.43 × 10^–02^	**3.33 × 10**^**–05**^
	Std	1.07 × 10^–01^	3.18 × 10^–03^	3.05 × 10^–02^	3.43 × 10^–03^	3.05 × 10^–02^	3.43 × 10^–03^	**2.84 × 10**^**–03**^
DTLZ4	Mean	4.88 × 10^–03^	5.67 × 10^–03^	2.04 × 10^–01^	5.67 × 10^–03^	2.04 × 10^–01^	8.73 × 10^–03^	**6.73 × 10**^**–05**^
	Std	8.15 × 10^–04^	5.87 × 10^–04^	3.71 × 10^–02^	1.05 × 10^–03^	3.71 × 10^–02^	3.85 × 10^–03^	**2.70 × 10**^**–04**^
DTLZ5	Mean	**9.16 × 10**^**–05**^	4.71 × 10^–04^	1.74 × 10^–01^	0.26 × 10^–01^	1.74 × 10^–01^	3.91 × 10^–03^	1.07 × 10^–04^
	Std	4.86 × 10^–04^	5.07 × 10^–05^	4.06 × 10^–02^	1.87 × 10^–04^	4.06 × 10^–02^	7.06 × 10^–03^	**4.35 × 10**^**–05**^
DTLZ6	Mean	4.86 × 10^–04^	4.31 × 10^–04^	–	0.53 × 10^–01^	–	3.46 × 10^–02^	**2.91 × 10**^**–04**^
	Std	8.57 × 10^–05^	4.16 × 10^–05^	–	2.87 × 10^–04^	–	8.08 × 10^–02^	**3.72 × 10**^**–05**^
DTLZ7	Mean	1.63 × 10^–03^	3.07 × 10^–03^	5.47 × 10^–03^	1.26 × 10^–02^	5.47 × 10^–03^	3.22 × 10^–03^	**4.02 × 10**^**–04**^
	Std	6.86 × 10^–04^	1.16 × 10^–03^	9.92 × 10^–04^	2.57 × 10^–04^	9.92 × 10^–04^	1.42 × 10^–03^	**1.27 × 10**^**–04**^
WFG4	Mean	2.25 × 10^–02^	**1.65 × 10**^**–02**^	3.79 × 10^–02^	2.44 × 10^–02^	2.56 × 10^–02^	3.90 × 10^–02^	1.68 × 10^–02^
	Std	5.38 × 10^–03^	9.05 × 10^–03^	3.51 × 10^–03^	6.87 × 10^–03^	1.16 × 10^–03^	1.63 × 10^–03^	**1.08 × 10**^**–03**^
WFG5	Mean	7.02 × 10^–03^	7.57 × 10^–03^	1.60 × 10^–02^	7.18 × 10^–03^	1.85 × 10^–02^	1.41 × 10^–02^	**6.52 × 10**^**–03**^
	Std	1.57 × 10^–04^	1.25 × 10^–04^	4.42 × 10^–03^	5.99 × 10^–04^	3.19 × 10^–03^	9.30 × 10^–04^	**6.92 × 10**^**–05**^
WFG6	Mean	5.19 × 10^–03^	1.02 × 10^–02^	2.57 × 10^–02^	9.66 × 10^–03^	2.48 × 10^–02^	3.49 × 10^–02^	**9.34 × 10**^**–04**^
	Std	4.83 × 10^–03^	3.82 × 10^–03^	3.89 × 10^–03^	5.41 × 10^–03^	**1.17 × 10**^**–03**^	1.52 × 10^–03^	1.39 × 10^–03^
WFG7	Mean	2.17 × 10^–02^	2.06 × 10^–02^	7.37 × 10^–02^	2.14 × 10^–02^	3.46 × 10^–02^	4.85 × 10^–02^	**1.08 × 10**^**–02**^
	Std	1.99 × 10^–03^	1.74 × 10^–03^	4.86 × 10^–03^	6.03 × 10^–03^	1.47 × 10^–03^	1.84 × 10^–03^	**1.46 × 10**^**–03**^
WFG8	Mean	6.77 × 10^–02^	8.41 × 10^–02^	6.83 × 10^–02^	8.37 × 10^–02^	4.44 × 10^–02^	6.42 × 10^–02^	**3.12 × 10**^**–02**^
	Std	1.53 × 10^–03^	2.80 × 10^–03^	4.53 × 10^–03^	1.04 × 10^–03^	2.09 × 10^–03^	2.20 × 10^–03^	**8.83 × 10**^**–04**^

Significant values are in bold.

**Table 4:** The IGD values obtained by all algorithms.

Problem		LMEA	WOF	MOSMA	DGEA	MOAHA	MOSPO	NSCSO
Bi-objective test function								
ZDT1	Mean	5.33 × 10^–03^	4.98 × 10^–03^	8.50 × 10^–03^	4.98 × 10^–03^	6.20 × 10^–03^	1.05 × 10^–02^	**4.93 × 10**^**–03**^
	Std	1.00 × 10^–03^	4.19 × 10^–04^	5.46 × 10^–04^	3.18 × 10^–04^	4.75 × 10^–04^	1.28 × 10^–03^	**2.58 × 10**^**–04**^
ZDT2	Mean	5.04 × 10^–03^	5.40 × 10^–03^	8.62 × 10^–03^	5.41 × 10^–03^	5.11 × 10^–03^	1.09 × 10^–02^	**4.91 × 10**^**–03**^
	Std	6.79 × 10^–04^	2.10 × 10^–03^	5.12 × 10^–04^	7.15 × 10^–04^	3.77 × 10^–04^	7.91 × 10^–04^	**2.52 × 10**^**–04**^
ZDT3	Mean	5.95 × 10^–03^	6.21 × 10^–03^	8.24 × 10^–03^	1.02 × 10^–02^	**4.44 × 10**^**–03**^	1.36 × 10^–02^	5.28 × 10^–03^
	Std	5.74 × 10^–04^	9.00 × 10^–04^	3.79 × 10^–04^	1.81 × 10^–03^	1.87 × 10^–04^	1.61 × 10^–02^	**1.75 × 10**^**–04**^
ZDT4	Mean	5.00 × 10^–03^	–	8.28 × 10^–03^	9.95 × 10^–03^	4.94 × 10^–03^	–	**4.89 × 10**^**–03**^
	Std	1.94 × 10^–03^	–	5.03 × 10^–04^	5.28 × 10^–04^	**7.86 × 10**^**–05**^	–	2.91 × 10^–04^
ZDT6	Mean	4.61 × 10^–03^	4.13 × 10^–03^	6.02 × 10^–02^	4.91 × 10^–03^	6.64 × 10^–03^	1.19 × 10^–02^	**3.18 × 10**^**–03**^
	Std	1.58 × 10^–03^	8.18 × 10^–04^	1.61 × 10^–02^	5.78 × 10^–04^	2.54 × 10^–03^	3.28 × 10^–01^	**5.73 × 10**^**–04**^
Three-objective test function								
DTLZ2	Mean	7.36 × 10^–02^	6.82 × 10^–02^	4.65 × 10^–01^	6.80 × 10^–02^	8.47 × 10^–02^	1.29 × 10^–01^	**6.49 × 10**^**–02**^
	Std	3.58 × 10^–03^	2.99 × 10^–03^	**1.25 × 10**^**–03**^	2.19 × 10^–03^	4.14 × 10^–03^	6.72 × 10^–03^	2.46 × 10^–03^
DTLZ4	Mean	6.36 × 10^–02^	8.62 × 10^–02^	4.20 × 10^–01^	8.62 × 10^–02^	7.79 × 10^–02^	9.31 × 10^–02^	**6.12 × 10**^**–02**^
	Std	1.82 × 10^–02^	5.56 × 10^–03^	**2.05 × 10**^**–03**^	4.60 × 10^–03^	4.34 × 10^–03^	6.85 × 10^–03^	4.17 × 10^–03^
DTLZ5	Mean	4.53 × 10^–03^	1.62 × 10^–02^	3.47 × 10^–01^	6.88 × 10^–02^	7.97 × 10^–03^	4.41 × 10^–02^	**5.41 × 10**^**–03**^
	Std	1.53 × 10^–04^	2.46 × 10^–04^	1.05 × 10^–04^	9.72 × 10^–05^	8.00 × 10^–04^	3.82 × 10^–03^	**2.22 × 10**^**–05**^
DTLZ6	Mean	5.48 × 10^–03^	2.29 × 10^–02^	–	6.51 × 10^–02^	7.04 × 10^–03^	1.16 × 10^–02^	**5.46 × 10**^**–03**^
	Std	8.15 × 10^–04^	3.51 × 10^–04^	–	1.00 × 10^–03^	**1.00 × 10**^**–04**^	1.10 × 10^–03^	3.72 × 10^–04^
DTLZ7	Mean	5.94 × 10^–02^	7.63 × 10^–02^	1.67 × 10^–01^	1.48 × 10^–01^	8.01 × 10^–02^	1.03 × 10^–01^	**4.33 × 10**^**–02**^
	Std	1.78 × 10^–01^	1.85 × 10^–02^	1.69 × 10^–02^	4.39 × 10^–02^	1.73 × 10^–02^	1.05 × 10^–02^	**5.41 × 10**^**–03**^
WFG4	Mean	3.13 × 10^–01^	5.20 × 10^–01^	4.76 × 10^–01^	5.35 × 10^–01^	3.73 × 10^–01^	5.11 × 10^–01^	**3.07 × 10**^**–01**^
	Std	3.88 × 10^–02^	1.70 × 10^–02^	2.79 × 10^–02^	**8.51 × 10**^**–03**^	1.49 × 10^–02^	2.89 × 10^–02^	9.89 × 10^–03^
WFG5	Mean	2.21 × 10^–01^	2.29 × 10^–01^	3.27 × 10^–01^	2.45 × 10^–01^	2.98 × 10^–01^	3.54 × 10^–01^	**1.81 × 10**^**–02**^
	Std	4.12 × 10^–02^	1.32 × 10^–02^	3.48 × 10^–02^	3.70 × 10^–02^	2.04 × 10^–02^	1.88 × 10^–02^	**8.80 × 10**^**–03**^
WFG6	Mean	4.13 × 10^–01^	3.36 × 10^–01^	5.27 × 10^–01^	4.53 × 10^–01^	4.23 × 10^–01^	4.91 × 10^–01^	**2.69 × 10**^**–01**^
	Std	1.11 × 10^–02^	**5.27 × 10**^**–03**^	6.53 × 10^–02^	2.79 × 10^–02^	3.29 × 10^–02^	8.49 × 10^–03^	8.63 × 10^–03^
WFG7	Mean	4.02 × 10^–01^	5.21 × 10^–01^	7.01 × 10^–01^	2.92 × 10^–01^	4.19 × 10^–01^	5.27 × 10^–01^	**2.69 × 10**^**–01**^
	Std	1.37 × 10^–02^	1.51 × 10^–02^	3.12 × 10^–02^	1.95 × 10^–02^	1.95 × 10^–02^	2.82 × 10^–02^	**9.34 × 10**^**–03**^
WFG8	Mean	6.24 × 10^–01^	1.07 × 10^+00^	8.73 × 10^–01^	7.47 × 10^–01^	4.98 × 10^–01^	6.73 × 10^–01^	**4.01 × 10**^**–01**^
	Std	1.50 × 10^–02^	2.18 × 10^–02^	4.27 × 10^–02^	9.08 × 10^–03^	1.32 × 10^–02^	3.64 × 10^–02^	**8.19 × 10**^**–03**^

Significant values are in bold.

**Table 5:** The SP values obtained by all algorithms.

Problem		LMEA	WOF	MOSMA	DGEA	MOAHA	MOSPO	NSCSO
Bi-objective test function								
ZDT1	Mean	5.28 × 10^–01^	2.89 × 10^–01^	1.04 × 10^–02^	2.89 × 10^–01^	3.06 × 10^–02^	1.04 × 10^–02^	6.95 × 10^–03^
	Std	3.18 × 10^–04^	1.03 × 10^–03^	9.44 × 10^–04^	4.02 × 10^–04^	**2.98 × 10**^**–04**^	1.26 × 10^–03^	4.98 × 10^–04^
ZDT2	Mean	2.46 × 10^–01^	1.41 × 10^–01^	9.92 × 10^–03^	1.41 × 10^–01^	9.30 × 10^–03^	9.01 × 10^–02^	**7.31 × 10**^**–03**^
	Std	7.62 × 10^–03^	6.08 × 10^–04^	1.00 × 10^–03^	4.71 × 10^–03^	8.80 × 10^–04^	1.58 × 10^–03^	**5.98 × 10**^**–04**^
ZDT3	Mean	3.10 × 10^–01^	8.49 × 10^–01^	2.21 × 10^–04^	5.29 × 10^–01^	4.06 × 10^–03^	1.02 × 10^–02^	**1.77 × 10**^**–04**^
	Std	**1.18 × 10**^**–05**^	1.07 × 10^–04^	1.26 × 10^–05^	1.61 × 10^–04^	3.88 × 10^–04^	1.36 × 10^–03^	1.44 × 10^–05^
ZDT4	Mean	4.64 × 10^–01^	–	9.91 × 10^–03^	2.89 × 10^–01^	3.32 × 10^–03^	–	**1.96 × 10**^**–04**^
	Std	5.81 × 10^–05^	–	4.69 × 10^–04^	6.55 × 10^–05^	1.48 × 10^–04^	–	**4.71 × 10**^**–05**^
ZDT6	Mean	2.34 × 10^–01^	1.03 × 10^–01^	2.27 × 10^–02^	1.37 × 10^–01^	2.47 × 10^–02^	6.42 × 10^–03^	**5.93 × 10**^**–03**^
	Std	1.38 × 10^–03^	1.92 × 10^–03^	2.56 × 10^–02^	1.96 × 10^–03^	2.52 × 10^–01^	2.93 × 10^–03^	**5.83 × 10**^**–04**^
Three-objective test function								
DTLZ2	Mean	8.47 × 10^–02^	1.73 × 10^–01^	2.90 × 10^–01^	1.73 × 10^–01^	6.34 × 10^–02^	7.99 × 10^–02^	**2.65 × 10**^**–03**^
	Std	4.21 × 10^–04^	1.08 × 10^–04^	5.73 × 10^–02^	9.07 × 10^–05^	5.58 × 10^–03^	8.38 × 10^–03^	**6.93 × 10**^**–05**^
DTLZ4	Mean	1.18 × 10^–01^	1.77 × 10^–01^	3.33 × 10^–01^	1.77 × 10^–01^	7.41 × 10^–02^	5.11 × 10^–02^	**1.82 × 10**^**–02**^
	Std	5.07 × 10^–03^	7.62 × 10^–03^	1.48 × 10^–01^	5.74 × 10^–03^	6.07 × 10^–03^	1.56 × 10^–02^	**2.17 × 10**^**–03**^
DTLZ5	Mean	2.89 × 10^–01^	8.80 × 10^–01^	2.73 × 10^–01^	6.09 × 10^–01^	8.96 × 10^–03^	3.20 × 10^–02^	**8.45 × 10**^**–03**^
	Std	1.68 × 10^–03^	**4.92 × 10**^**–04**^	5.02 × 10^–02^	5.12 × 10^–03^	1.17 × 10^–03^	6.71 × 10^–03^	**8.07 × 10**^**–04**^
DTLZ6	Mean	3.09 × 10^–01^	1.28 × 10^+00^	–	6.82 × 10^–01^	**3.93 × 10**^**–03**^	2.27 × 10^–02^	8.90 × 10^–03^
	Std	7.34 × 10^–03^	1.90 × 10^–03^	–	1.08 × 10^–03^	**6.16 × 10**^**–04**^	4.22 × 10^–02^	7.69 × 10^–04^
DTLZ7	Mean	3.11 × 10^–01^	5.43 × 10^–01^	3.41 × 10^–02^	3.42 × 10^–01^	7.77 × 10^–02^	9.09 × 10^–02^	**3.32 × 10**^**–02**^
	Std	4.83 × 10^–03^	1.49 × 10^–02^	6.13 × 10^–03^	2.20 × 10^–02^	7.21 × 10^–03^	7.79 × 10^–03^	**2.14 × 10**^**–03**^
WFG4	Mean	3.67 × 10^–01^	2.86 × 10^–01^	2.91 × 10^–01^	2.97 × 10^–01^	2.47 × 10^–01^	2.39 × 10^–01^	**2.15 × 10**^**–01**^
	Std	6.15 × 10^–02^	3.73 × 10^–02^	2.24 × 10^–02^	1.80 × 10^–03^	2.17 × 10^–02^	2.60 × 10^–02^	**1.92 × 10**^**–02**^
WFG5	Mean	2.35 × 10^–01^	2.90 × 10^–01^	3.27 × 10^–01^	3.01 × 10^–01^	1.85 × 10^–01^	4.40 × 10^–02^	**1.98 × 10**^**–02**^
	Std	1.86 × 10^–03^	**1.58 × 10**^**–03**^	3.47 × 10^–02^	1.86 × 10^–03^	3.12 × 10^–03^	3.93 × 10^–03^	**1.74 × 10**^**–03**^
WFG6	Mean	2.25 × 10^–01^	2.92 × 10^–01^	2.72 × 10^–01^	3.02 × 10^–01^	2.48 × 10^–01^	2.78 × 10^–01^	**2.07 × 10**^**–01**^
	Std	5.37 × 10^–03^	3.28 × 10^–03^	5.55 × 10^–02^	1.87 × 10^–03^	1.95 × 10^–02^	1.68 × 10^–02^	**1.63 × 10**^**–03**^
WFG7	Mean	2.12 × 10^–01^	2.86 × 10^–01^	2.61 × 10^–01^	2.96 × 10^–01^	2.43 × 10^–01^	2.38 × 10^–01^	**2.07 × 10**^**–01**^
	Std	1.84 × 10^–02^	1.64 × 10^–02^	2.01 × 10^–02^	1.47 × 10^–02^	1.64 × 10^–02^	2.06 × 10^–02^	**1.02 × 10**^**–02**^
WFG8	Mean	2.93 × 10^–01^	2.95 × 10^–01^	3.97 × 10^–01^	3.30 × 10^–01^	2.32 × 10^–01^	2.54 × 10^–01^	**2.17 × 10**^**–01**^
	Std	4.15 × 10^–02^	3.15 × 10^–02^	1.05 × 10^–01^	3.71 × 10^–01^	3.86 × 10^–02^	2.81 × 10^–02^	**2.10 × 10**^**–02**^

Significant values are in bold.

**Table 6:** The MS values obtained by all algorithms.

Problem		LMEA	WOF	MOSMA	DGEA	MOAHA	MOSPO	NSCSO
Bi-objective test function								
ZDT1	Mean	1.79 × 10^–02^	9.99 × 10^–02^	1.47 × 10^+00^	9.85 × 10^–02^	5.39 × 10^–01^	1.57 × 10^+00^	**3.97 × 10**^**–01**^
	Std	5.03 × 10^–02^	4.14 × 10^–02^	5.70 × 10^–02^	6.44 × 10^–02^	4.86 × 10^–02^	1.45 × 10^–01^	**3.87 × 10**^**–02**^
ZDT2	Mean	6.46 × 10^–01^	4.21 × 10^–01^	1.45 × 10^+00^	4.21 × 10^–01^	6.03 × 10^–01^	1.67 × 10^+00^	**4.14 × 10**^**–01**^
	Std	4.61 × 10^–02^	4.70 × 10^–02^	7.96 × 10^–02^	2.19 × 10^–02^	**2.29 × 10**^**–02**^	7.51 × 10^–02^	3.95 × 10^–02^
ZDT3	Mean	**3.38 × 10**^**–01**^	4.15 × 10^–01^	1.25 × 10^+00^	4.16 × 10^–01^	5.80 × 10^–01^	1.66 × 10^+00^	3.92 × 10^–01^
	Std	1.20 × 10^–01^	5.50 × 10^–02^	8.26 × 10^–02^	**4.16 × 10**^**–02**^	4.79 × 10^–02^	1.15 × 10^–01^	4.53 × 10^–02^
ZDT4	Mean	7.28 × 10^–01^	–	1.47 × 10^+00^	9.84 × 10^–01^	5.91 × 10^–01^	–	**4.06 × 10**^**–01**^
	Std	3.98 × 10^–02^	–	6.67 × 10^–02^	4.78 × 10^–02^	3.87 × 10^–02^	–	**3.59 × 10**^**–02**^
ZDT6	Mean	4.55 × 10^–01^	4.09 × 10^–01^	1.26 × 10^+00^	4.82 × 10^–01^	9.74 × 10^–01^	1.56 × 10^+00^	**3.99 × 10**^**–01**^
	Std	4.56 × 10^–02^	1.50 × 10^–01^	1.50 × 10^–01^	1.15 × 10^–01^	7.15 × 10^–01^	3.77 × 10^–01^	**4.58 × 10**^**–02**^
Three-objective test function								
DTLZ2	Mean	6.25 × 10^–01^	5.77 × 10^–02^	9.86 × 10^–01^	**5.72 × 10**^**–02**^	4.27 × 10^–01^	5.89 × 10^–01^	6.24 × 10^–02^
	Std	3.27 × 10^–02^	4.33 × 10^–02^	1.50 × 10^–01^	3.86 × 10^–02^	3.54 × 10^–02^	5.60 × 10^–02^	**3.22 × 10**^**–02**^
DTLZ4	Mean	2.29 × 10^–01^	5.68 × 10^–02^	1.24 × 10^+00^	5.72 × 10^–02^	6.73 × 10^–01^	1.41 × 10^+00^	**2.37 × 10**^**–02**^
	Std	1.06 × 10^–01^	8.29 × 10^–02^	1.37 × 10^–01^	1.32 × 10^–01^	6.71 × 10^–02^	9.25 × 10^–02^	**4.75 × 10**^**–02**^
DTLZ5	Mean	8.30 × 10^–01^	5.65 × 10^–01^	1.32 × 10^+00^	4.57 × 10^–01^	7.05 × 10^–01^	1.24 × 10^+00^	**4.07 × 10**^**–01**^
	Std	5.56 × 10^–02^	5.68 × 10^–02^	1.10 × 10^–01^	5.31 × 10^–02^	6.24 × 10^–02^	1.14 × 10^–01^	**4.52 × 10**^**–02**^
DTLZ6	Mean	8.45 × 10^–01^	7.43 × 10^–01^	–	8.60 × 10^–01^	5.01 × 10^–01^	1.72 × 10^+00^	**4.45 × 10**^**–01**^
	Std	1.64 × 10^–02^	1.84 × 10^–02^	–	9.93 × 10^–02^	1.26 × 10^–02^	1.38 × 10^–01^	**1.16 × 10**^**–02**^
DTLZ7	Mean	5.91 × 10^–01^	7.06 × 10^–01^	1.19 × 10^+00^	8.13 × 10^–01^	5.01 × 10^–01^	7.87 × 10^–01^	**9.08 × 10**^**–02**^
	Std	5.78 × 10^–02^	1.94 × 10^–01^	4.07 × 10^–02^	1.51 × 10^–01^	4.29 × 10^–02^	1.09 × 10^–01^	4.55 × 10^–02^
WFG4	Mean	1.26 × 10^–01^	2.15 × 10^–01^	5.89 × 10^–01^	2.75 × 10^–01^	4.19 × 10^–01^	5.16 × 10^–01^	**8.07 × 10**^**–02**^
	Std	3.15 × 10^–02^	2.85 × 10^–02^	3.53 × 10^–02^	3.04 × 10^–02^	2.68 × 10^–02^	3.32 × 10^–02^	**2.25 × 10**^**–02**^
WFG5	Mean	**1.67 × 10**^**–01**^	2.15 × 10^–01^	5.76 × 10^–01^	3.74 × 10^–01^	3.88 × 10^–01^	5.87 × 10^–01^	4.15 × 10^–01^
	Std	7.37 × 10^–02^	8.45 × 10^–02^	**1.69 × 10**^**–02**^	5.37 × 10^–02^	2.31 × 10^–02^	3.64 × 10^–02^	2.76 × 10^–02^
WFG6	Mean	4.48 × 10^–01^	5.15 × 10^–01^	7.87 × 10^–01^	5.74 × 10^–01^	4.23 × 10^–01^	5.95 × 10^–01^	**3.82 × 10**^**–01**^
	Std	1.53 × 10^–01^	3.67 × 10^–02^	7.57 × 10^–02^	1.42 × 10^–01^	3.29 × 10^–02^	4.62 × 10^–02^	**2.58 × 10**^**–02**^
WFG7	Mean	4.61 × 10^–01^	5.15 × 10^–01^	5.63 × 10^–01^	4.74 × 10^–01^	4.49 × 10^–01^	4.91 × 10^–01^	**3.76 × 10**^**–01**^
	Std	4.11 × 10^–02^	**1.40 × 10**^**–02**^	4.28 × 10^–02^	5.38 × 10^–02^	3.21 × 10^–02^	2.20 × 10^–01^	3.19 × 10^–02^
WFG8	Mean	8.32 × 10^–01^	1.20 × 10^+00^	7.74 × 10^–01^	6.81 × 10^–01^	4.87 × 10^–01^	4.72 × 10^–01^	**4.21 × 10**^**–01**^
	Std	1.93 × 10^–01^	**8.27 × 10**^**–02**^	1.19 × 10^–01^	2.35 × 10^–01^	3.76 × 10^–01^	2.68 × 10^–01^	2.16 × 10^–01^

Significant values are in bold.

**Figure 4:**
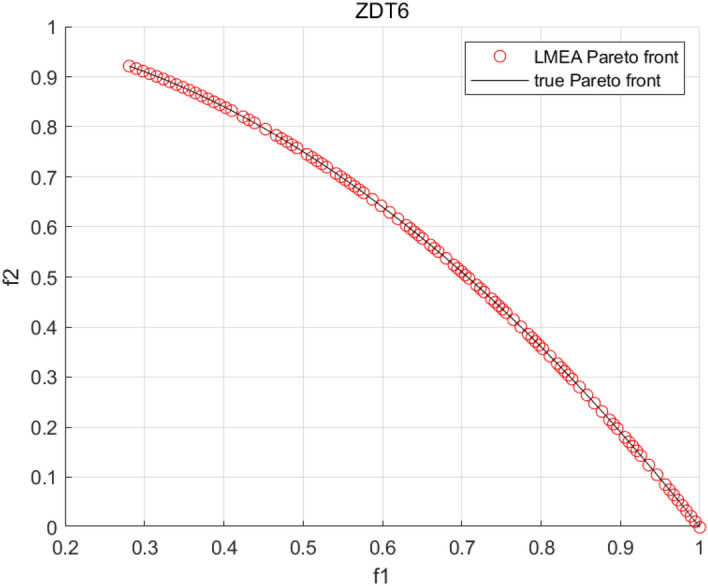
The Pareto front obtained by LMEA on ZDT6.

**Figure 5:**
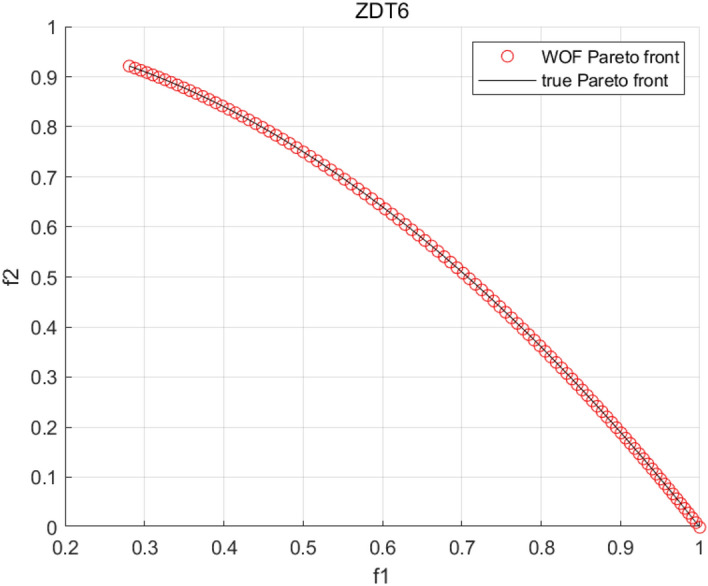
The Pareto front obtained by WOF on ZDT6.

**Figure 6:**
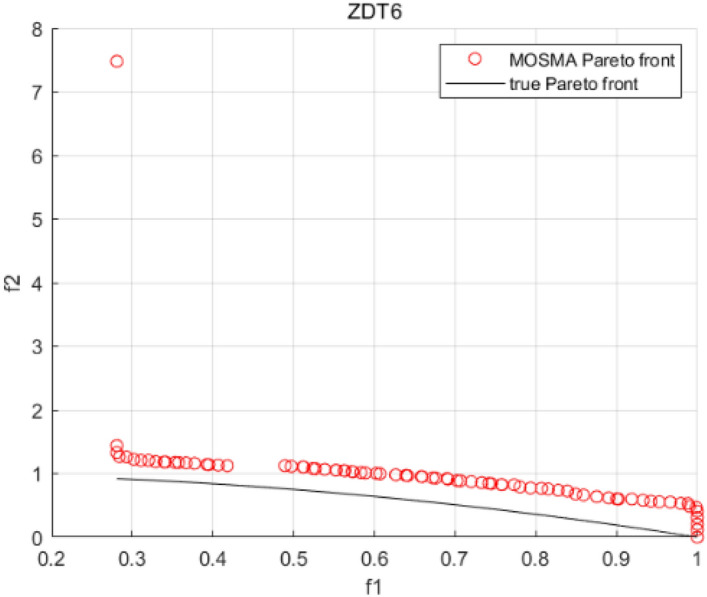
The Pareto front obtained by MOSMA on ZDT6**.**

**Figure 7:**
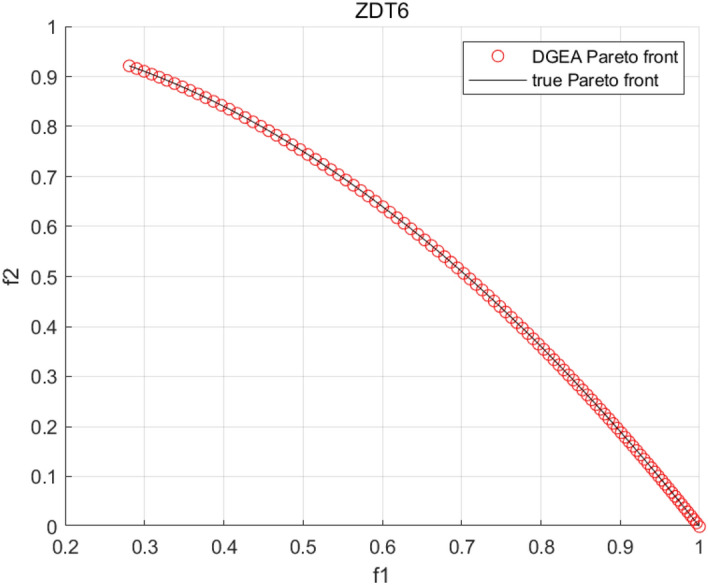
The Pareto front obtained DGEA by on ZDT6.

**Figure 8:**
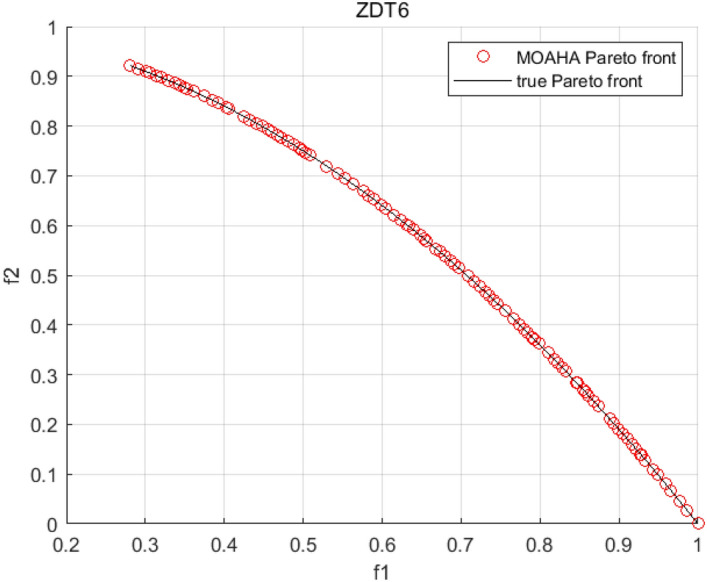
The Pareto front obtained by MOAHA on ZDT6**.**

**Figure 9:**
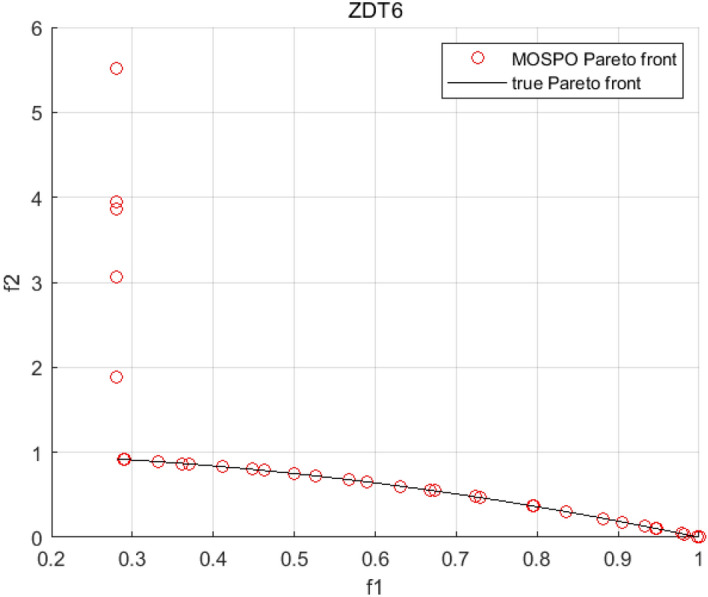
The Pareto front obtained by MOSPO on ZDT6.

**Figure 10:**
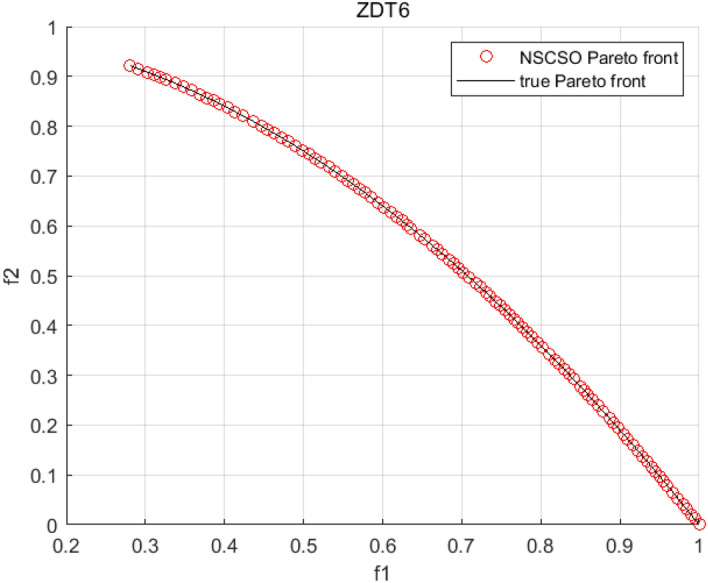
The Pareto front obtained by NSCSO on ZDT6.

**Figure 11:**
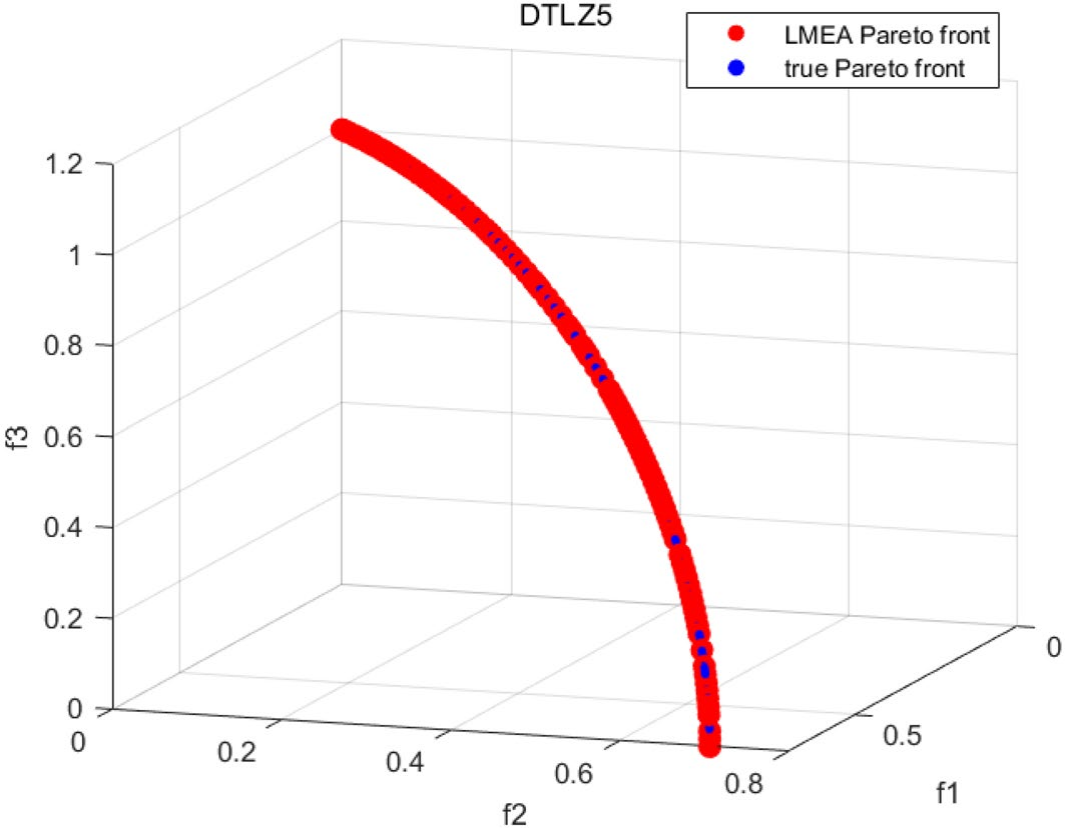
The Pareto front obtained by LMEA on DTLZ5.

**Figure 12:**
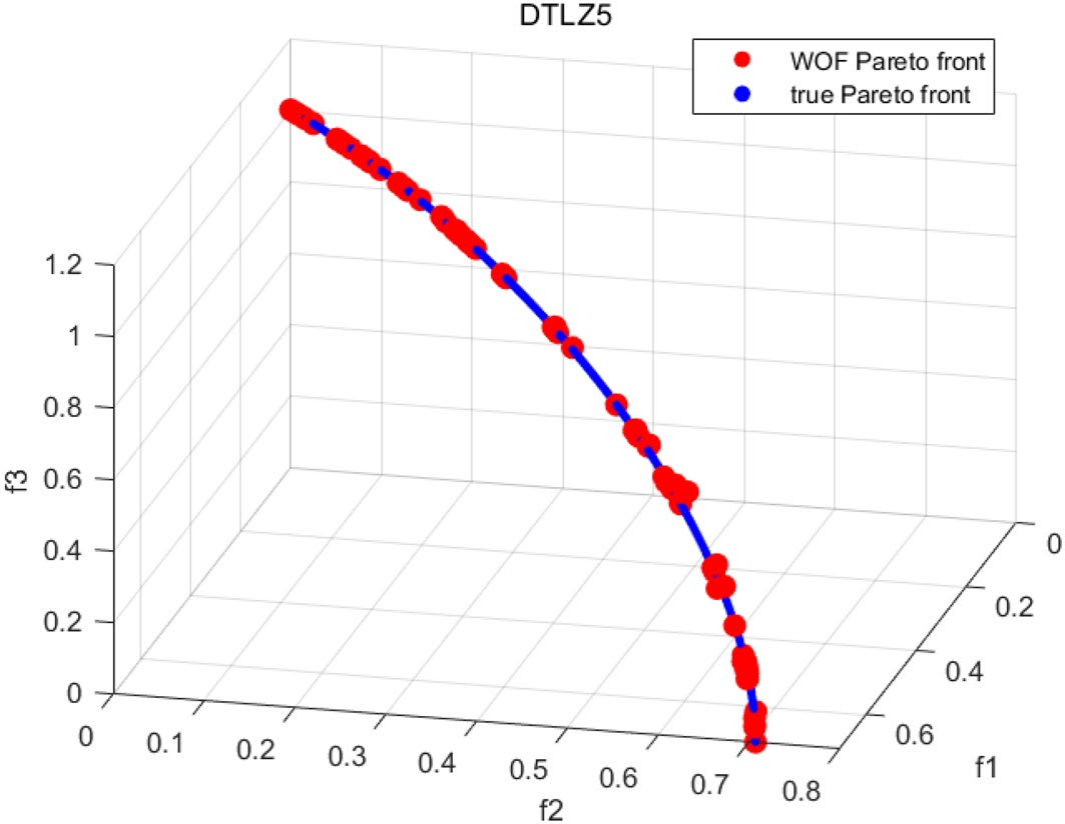
The Pareto front obtained by WOF on DTLZ5.

**Figure 13:**
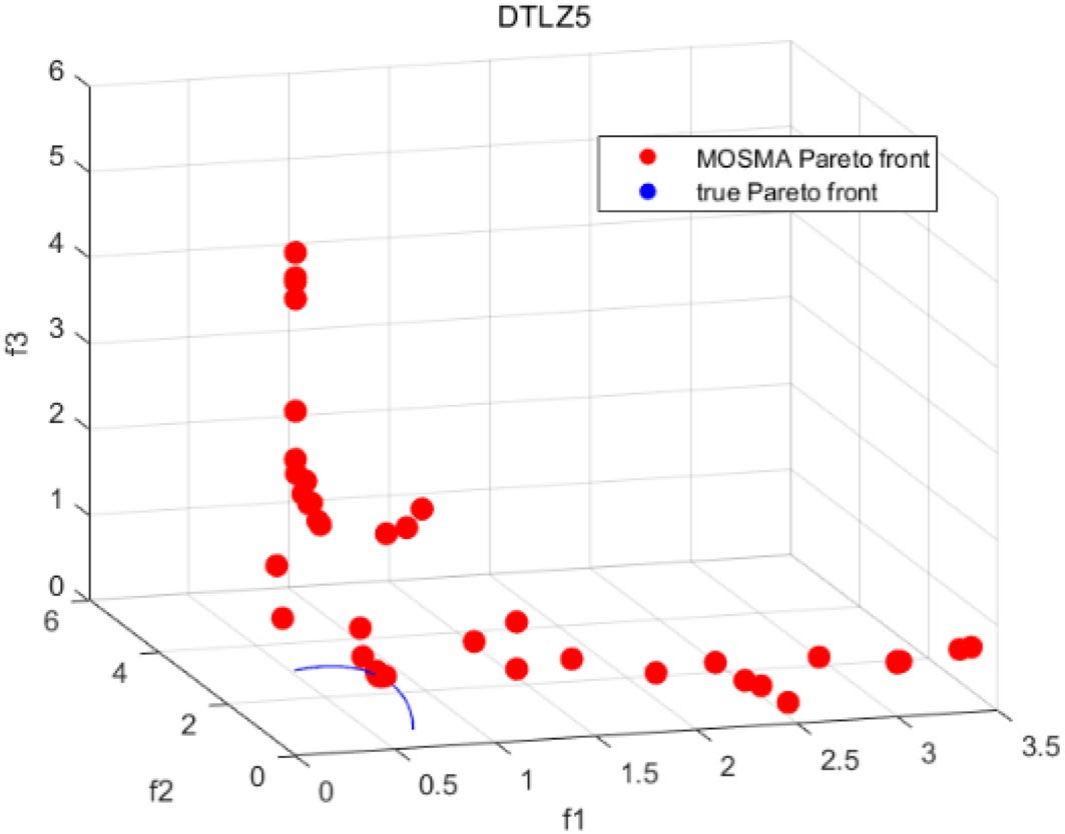
The Pareto front obtained by MOSMA on DTLZ5.

**Figure 14:**
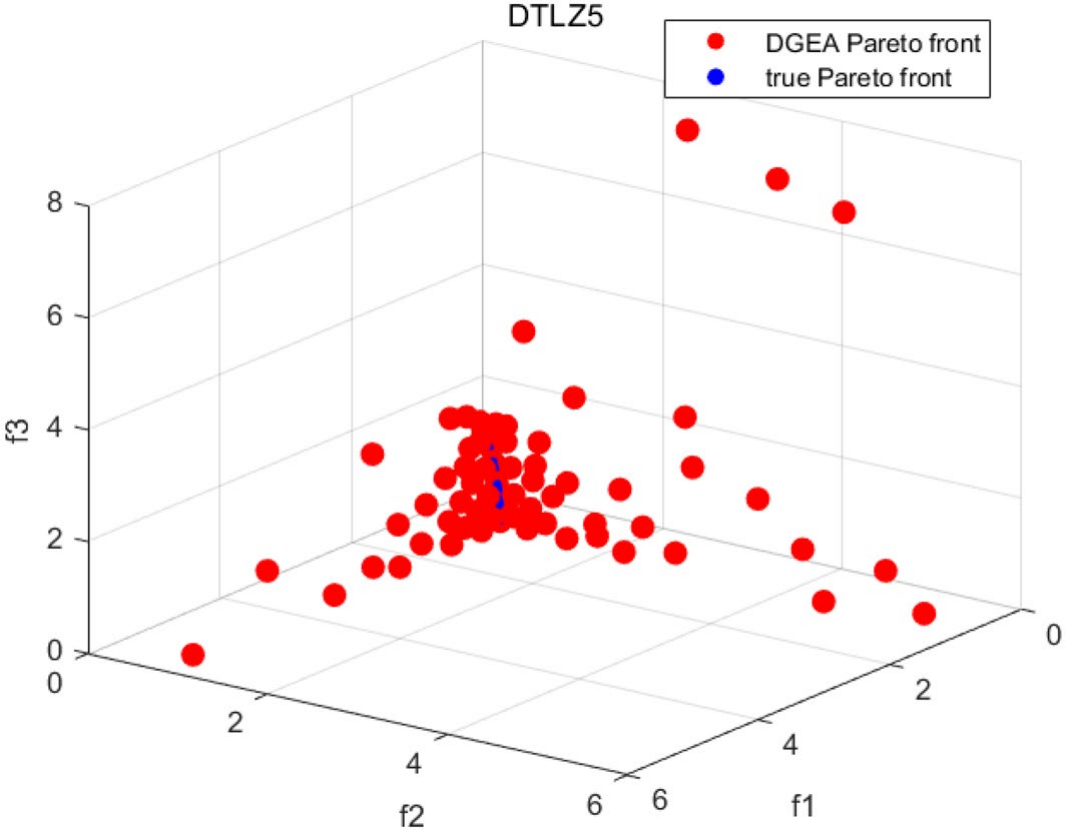
The Pareto front obtained by DGEA on DTLZ5.

**Figure 15:**
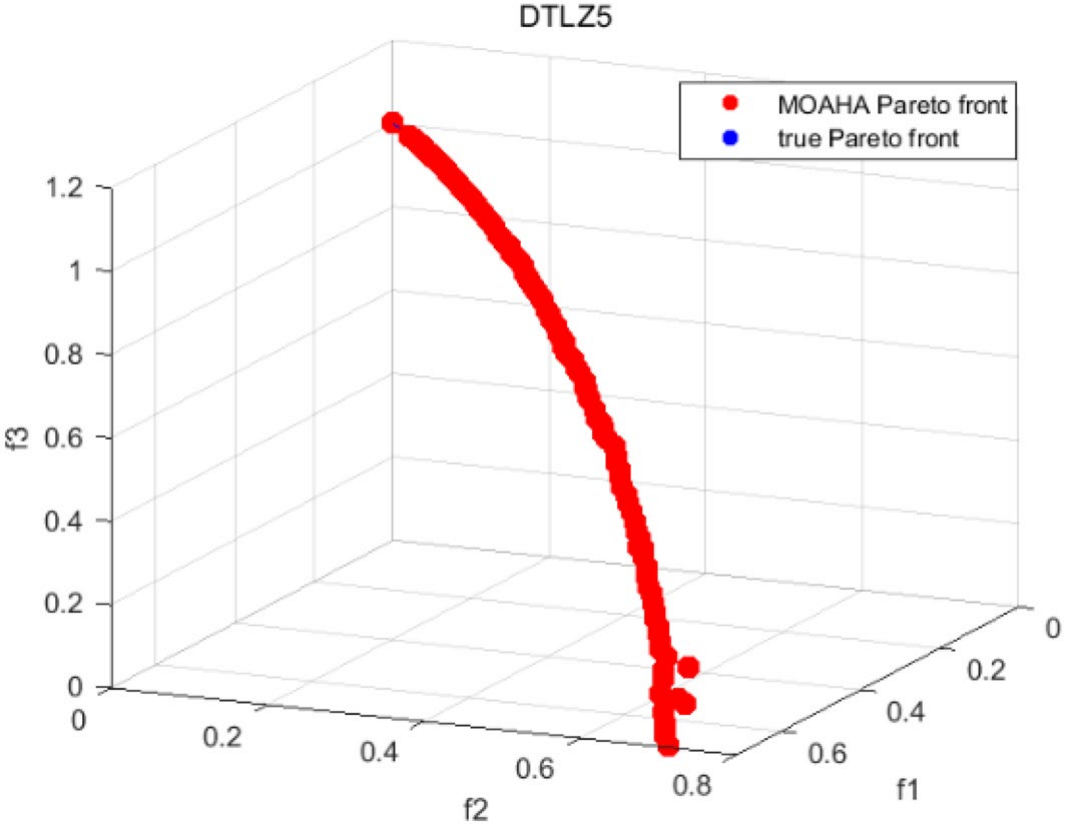
The Pareto front obtained by MOAHA on DTLZ5.

**Figure 16:**
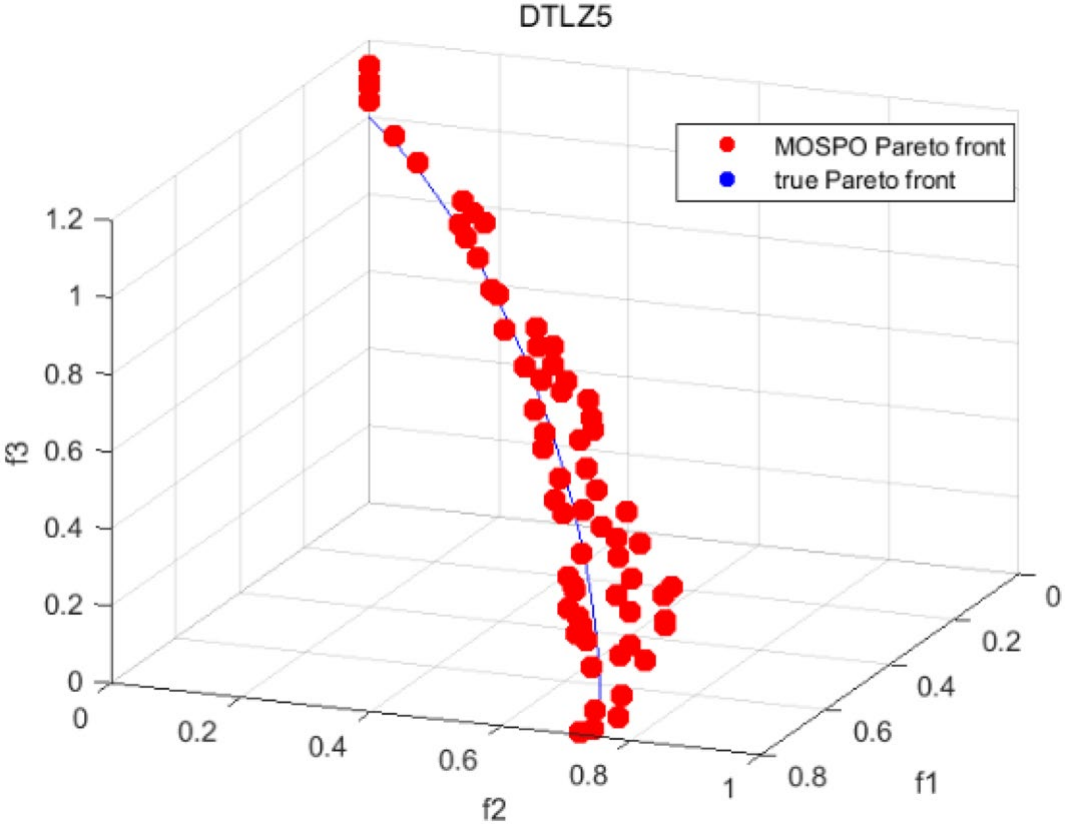
The Pareto front obtained by MOSPO on DTLZ5**.**

**Figure 17:**
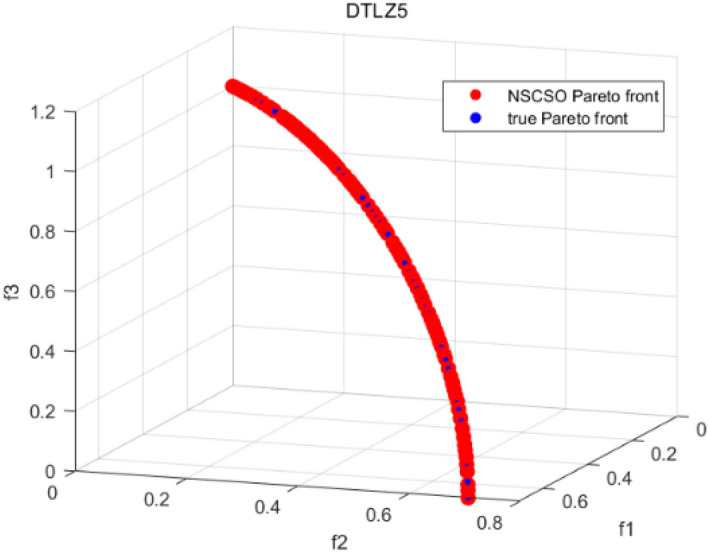
The Pareto front obtained by NSCSO on DTLZ5.

**Figure 18:**
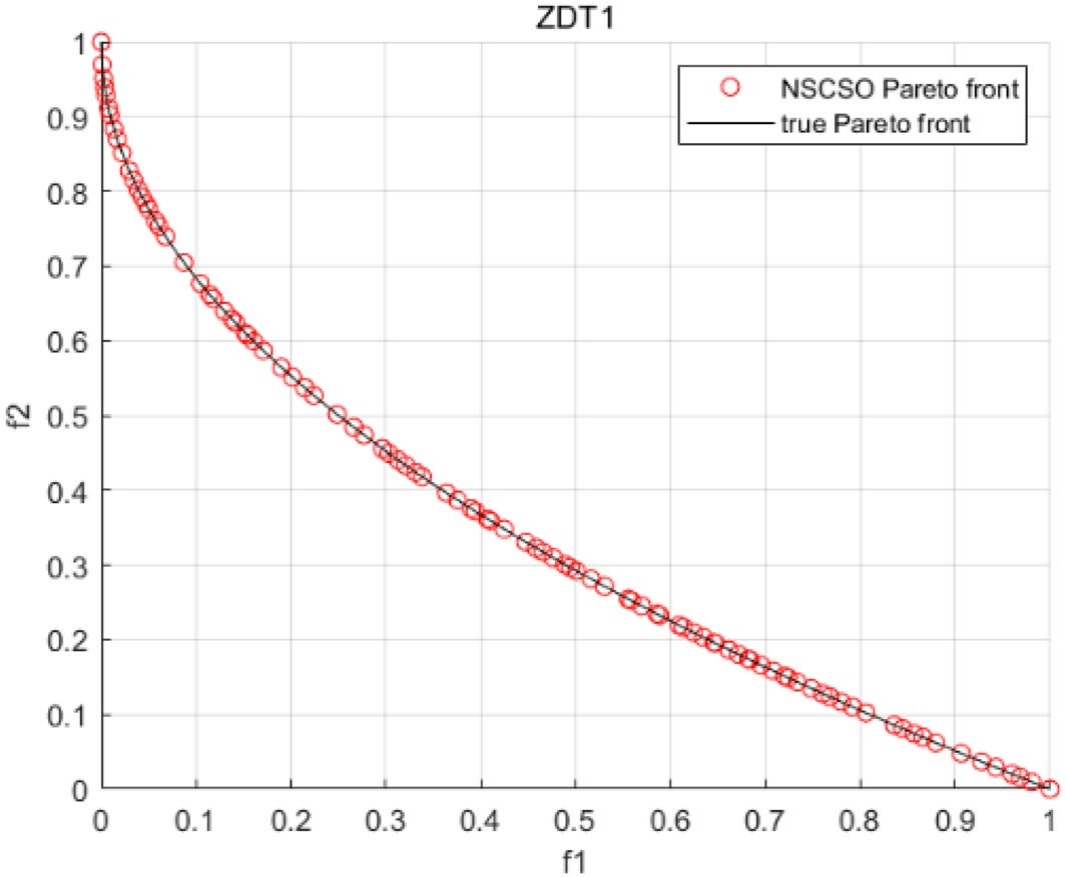
The Pareto front obtained by NSCSO on ZDT1.

**Figure 19:**
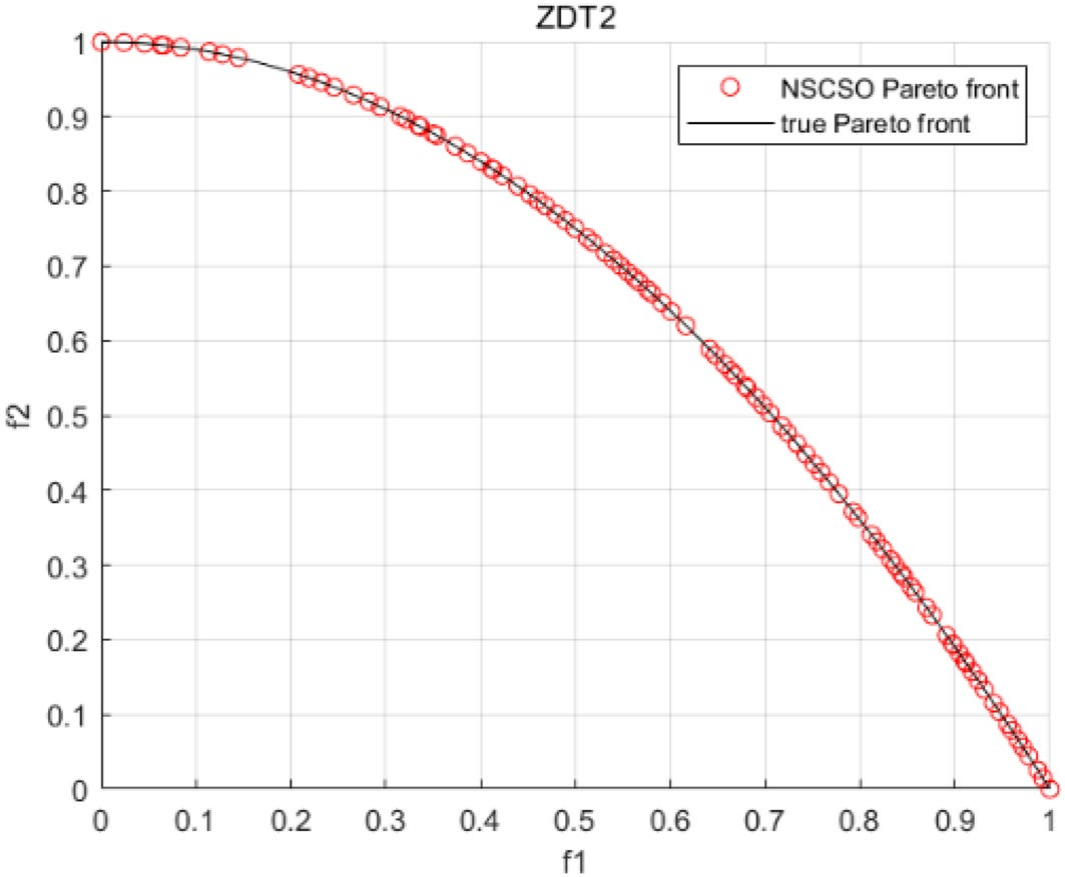
The Pareto front obtained by NSCSO on ZDT2.

**Figure 20:**
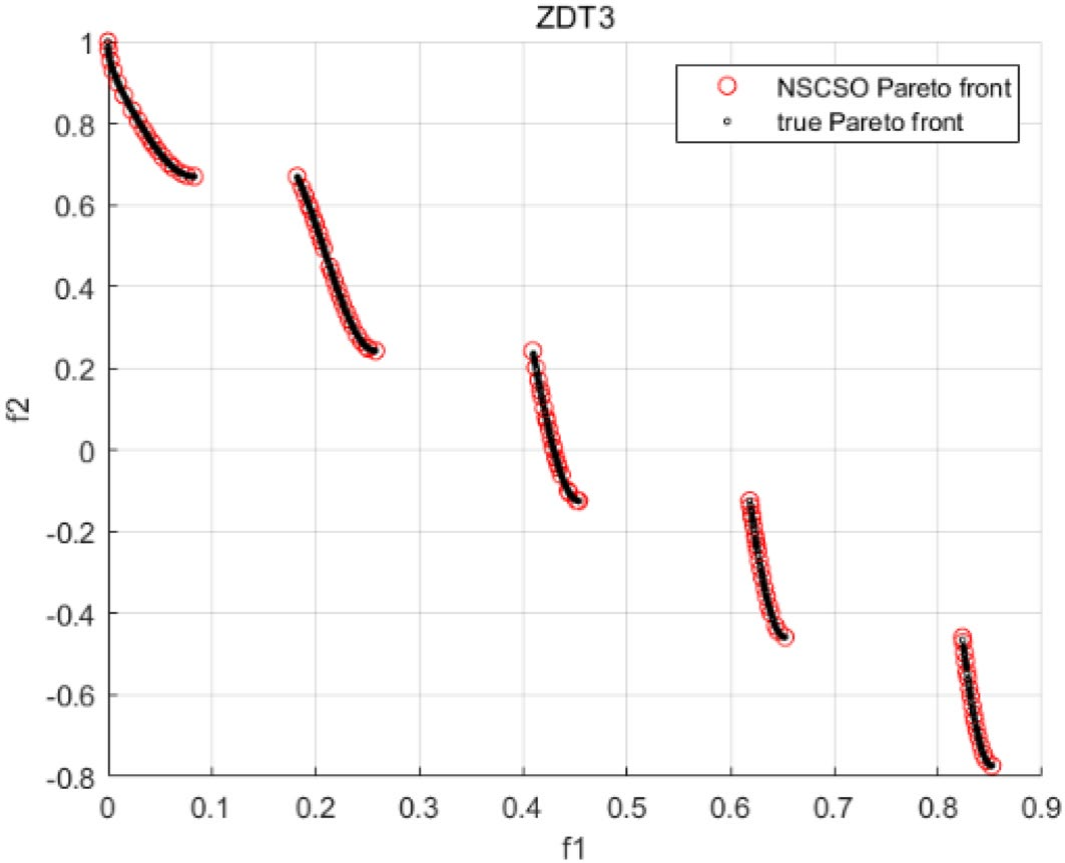
The Pareto front obtained by NSCSO on ZDT3.

**Figure 21:**
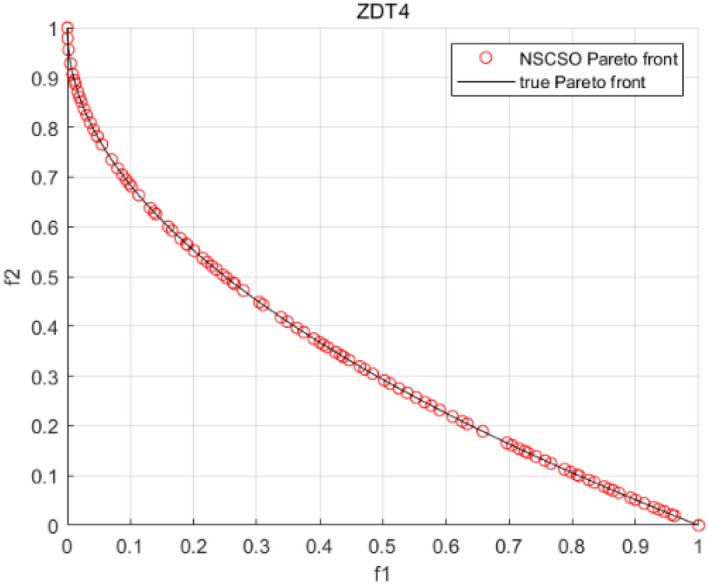
The Pareto front obtained by NSCSO on ZDT4.

**Figure 22:**
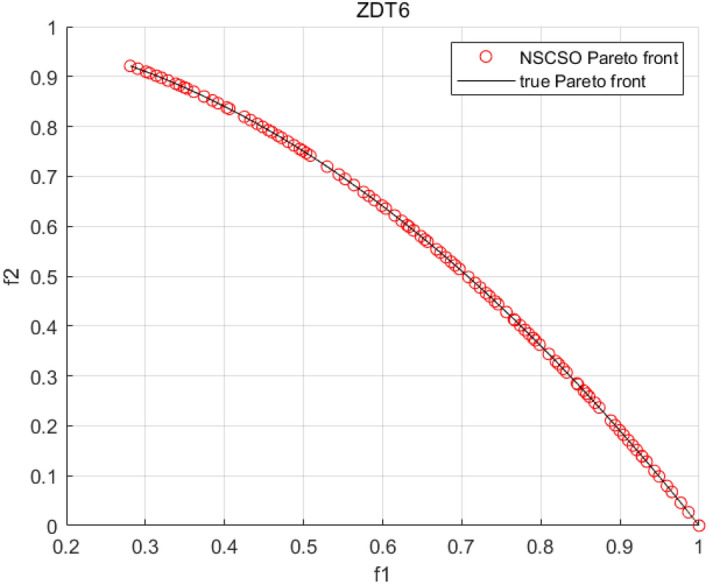
The Pareto front obtained by NSCSO on ZDT6.

**Figure 23:**
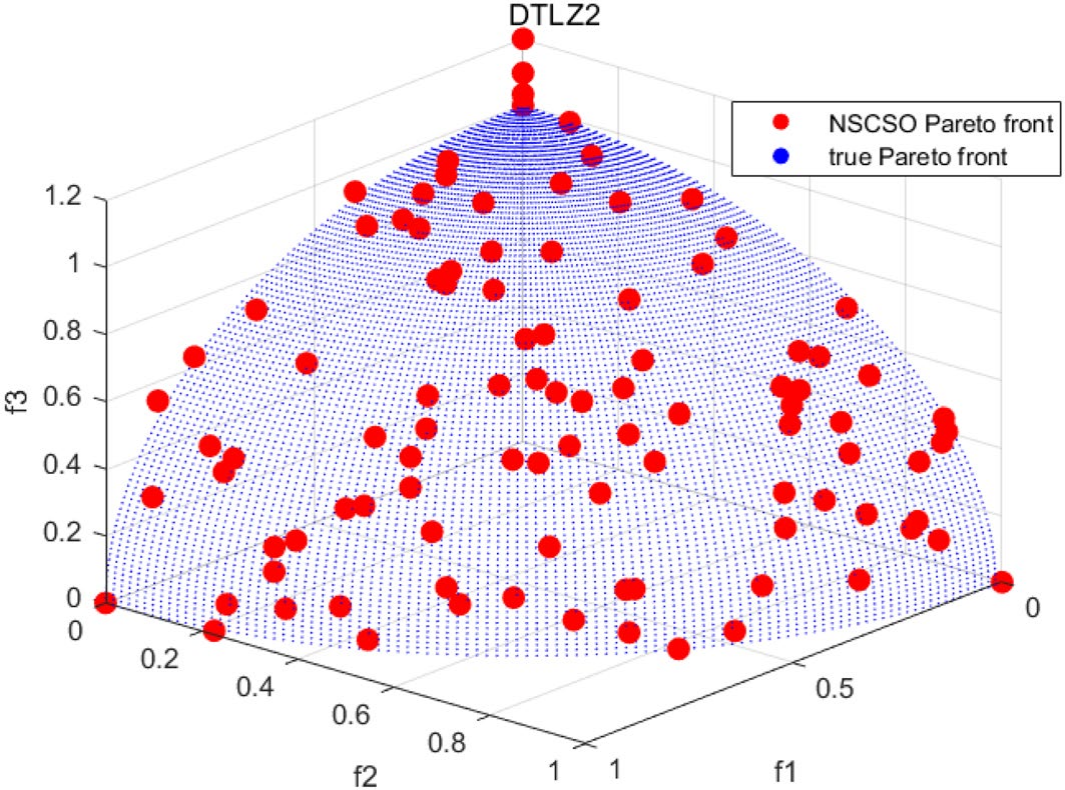
The Pareto front obtained by NSCSO on DTLZ2.

**Figure 24:**
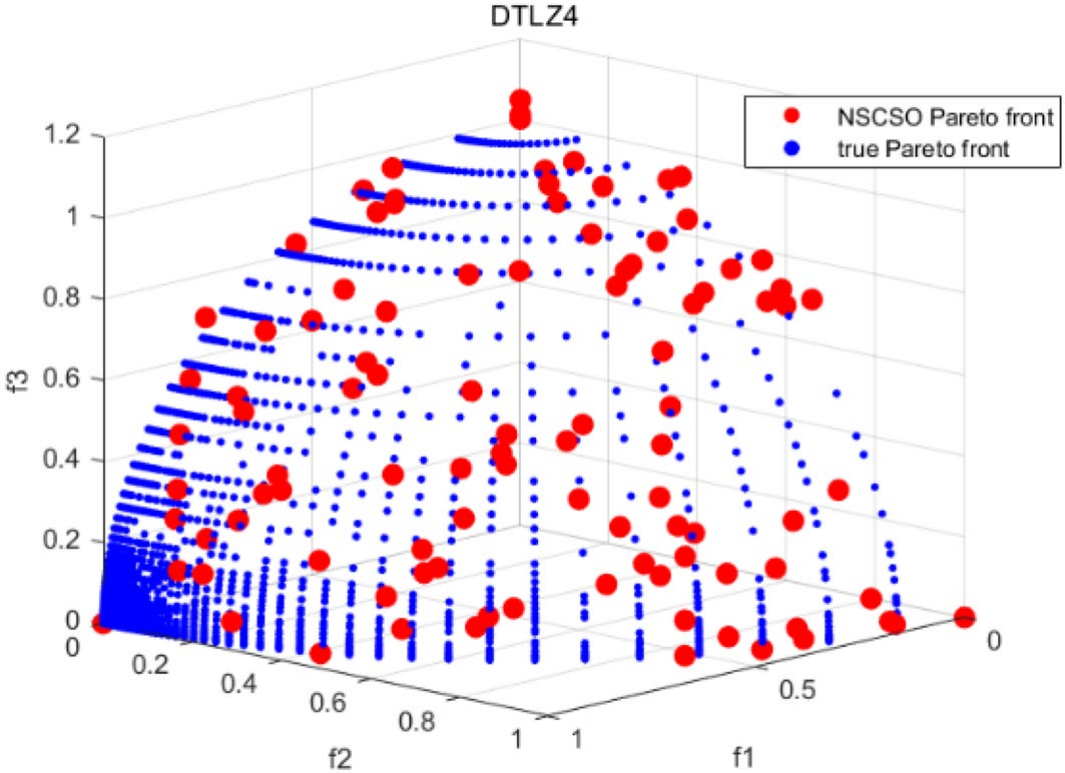
The Pareto front obtained by NSCSO on DTLZ4.

**Figure 25:**
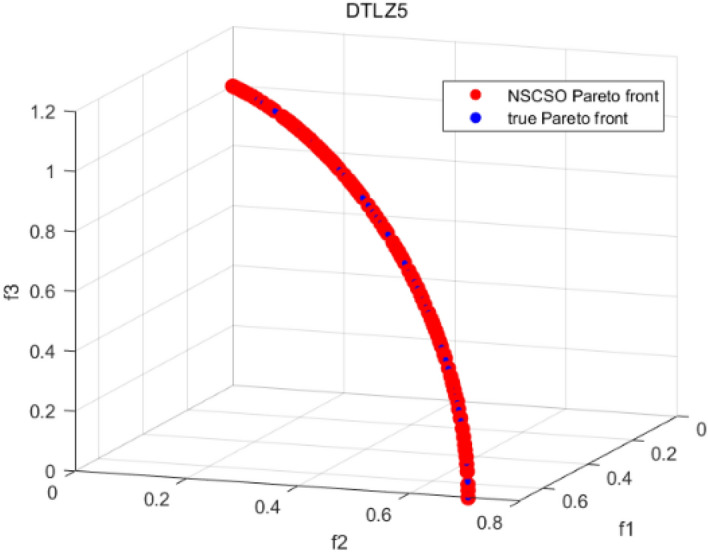
The Pareto front obtained by NSCSO on DTLZ5.

**Figure 26:**
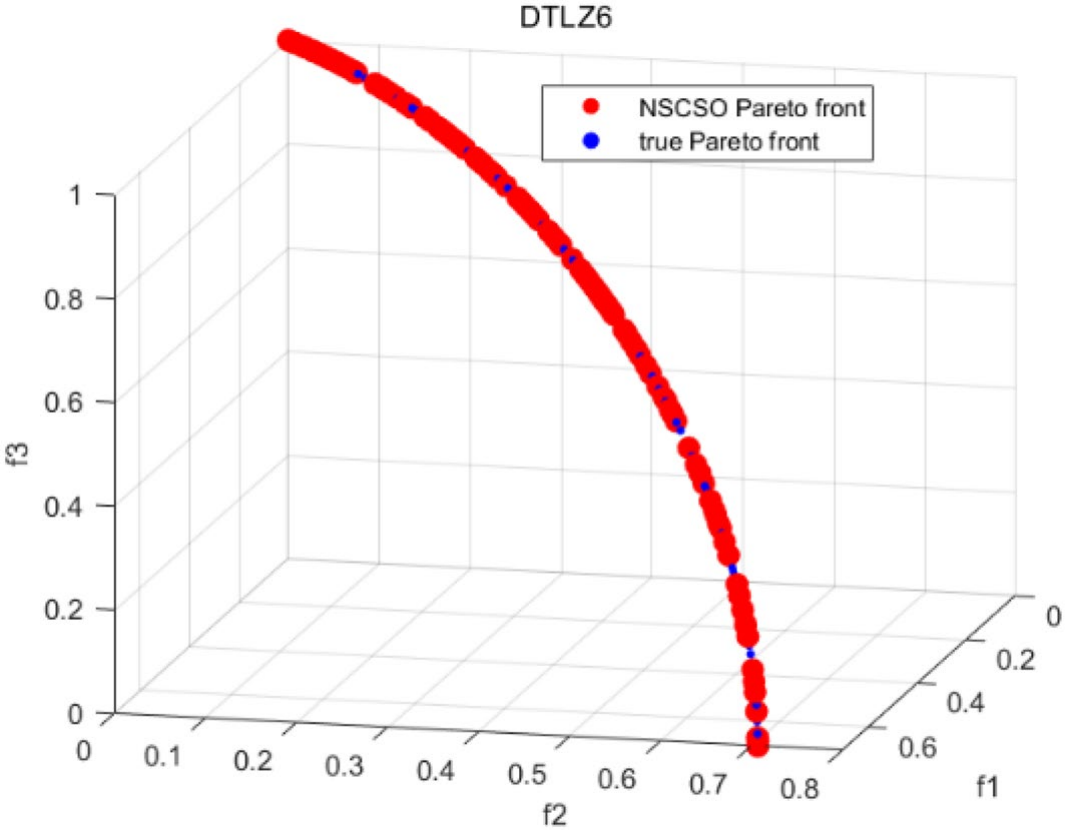
The Pareto front obtained by NSCSO on DTLZ6.

**Figure 27:**
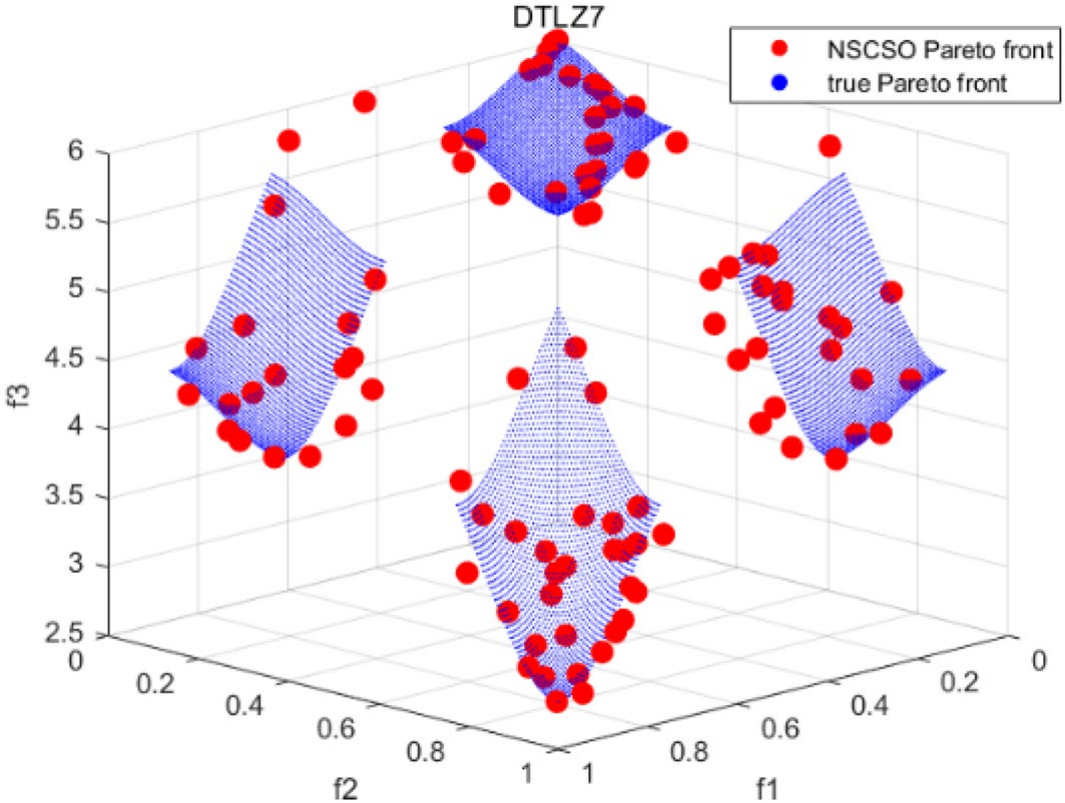
The Pareto front obtained by NSCSO on DTLZ7.

The GD values obtained by algorithms such as NSCSO are counted in Table 3, and the optimal value obtained in all results has been bolded. By comparing the data in Table 3, it can be known that in the ZDT test problem, the NSCSO algorithm obtains the minimum value in most of the test functions. In the ZDT1 benchmark function, the mean GD of WOF is better than NSCSO, but the difference between the two is very small. In ZDT3 and ZDT4, the results obtained by the MOSMA algorithm have better standard deviation values, but the difference between their mean values and those obtained by the NSCSO algorithm is relatively large, and the standard deviation values obtained by the NSCSO algorithm are within the acceptable range. ZDT4 is a highly multimodal function, and the WOF and MOSOP algorithms did not obtain the correct Pareto optimal solution in this algorithm, while the NSCSO algorithm was able to obtain the Pareto optimal solution with excellent convergence, and its GD value was much better than other algorithms. In the DTLZ testing problem, the NSCSO algorithm performed slightly worse than LMEA in the DTLZ5 test. In other problems, NSCSO obtained better Pareto optimal solutions than other algorithms, with the smallest standard deviation and greater stability. MOSMA and MOAHA were unable to stably obtain ideal Pareto optimal solutions in the testing of ZDT6. The solution difficulty of the WFG test set is higher than the other two test sets, but the NSCSO algorithm can still obtain more accurate Pareto optimal solutions in different test functions, it shows that the NSCSO algorithm can perform well in different test problems. The smaller the GD value, the better the convergence of the corresponding algorithm, and the more accurate the solution set obtained. Comparing the GD values obtained by different algorithms in Table 3, it can be seen that the GD value obtained by the NSCSO algorithm is small, which proves that it can find an excellent Pareto optimal solution when solving MOP, and has excellent convergence.

The IGD values obtained by algorithms such as NSCSO are counted in Table 4. In the ZDT test, the NSCSO algorithm performed slightly worse than the MOAHA algorithm in the ZDT3 test, ranking second. In other ZDT tests, the NSCSO algorithm achieved the best results among the seven algorithms in most of the tests. In the DTLZ testing problem, the mean of the NSCSO algorithm is also the smallest among all algorithms, and its standard deviation can reach E−03. Although the standard deviation does not rank first in some tests, it is still relatively small compared to the first place. In the WFG test function, the NSCSO algorithm also performs very well. In the testing of WFG5, only the NSCSO algorithm achieved 10^–02^, while the best of other algorithms only reached 10^–01^. While there are many similarities in the way IGD and GD are calculated, the GD index is more inclined to measure the convergence. IGD is a relatively comprehensive performance measure, which can not only detect the convergence of the obtained solution but also evaluate its diversity and extensiveness. Therefore, it can be concluded from the data in Table 4 that the NSCSO algorithm not only has excellent convergence but also the Pareto optimal solution obtained by it has excellent diversity and distribution.

The SP values obtained by algorithms such as NSCSO are counted in Table 5. In the ZDT testing problem, the NSCSO algorithm performed well and obtained better results than other algorithms. Although in ZDT3, the standard deviation was slightly inferior to LMEA, the SP obtained by the LMEA algorithm was far inferior to the NSCSO algorithm. In the DTLZ testing problem, except for DTLZ6, the NSCSO algorithm achieved first place, and its DTLZ6 results were also quite satisfactory. In the WFG test function, the NSCSO algorithm still obtained the best SP value. Since the SP metric mainly evaluates the distribution of the solutions obtained by the algorithm, the comparison of the SP indicates that the Pareto optimal solutions obtained by the NSCSO algorithm are more uniformly distributed in the multi-objective space compared with the other algorithms.

Table 6 records the MS results obtained by algorithms such as NSCSO. From the data in the table, it can be seen that in the ZDT test set, the mean of the NSCSO algorithm can obtain the best value among the seven algorithms in both the ZDT and DTLZ test sets. And although its standard deviation does not reach the first place in some functions, the difference is also very small. In the WFG test set, only the mean of WFG5 was worse than LMEA, ranking second. At the same time, the standard deviation of the WOF algorithm is better than that of the NSCSO algorithm, but in terms of mean, NSCSO obtains much better results than the WOF algorithm. MS value and SP value are both measures of the distributivity of the obtained solution set, and the MS value is mainly used to measure the coverage of the obtained solution to the true Pareto solution. According to the above description, the NSCSO algorithm can obtain a smaller MS value than other algorithms, so the Pareto optimal solution obtained by the NSCSO algorithm can cover the real Pareto optimal solution more widely than other algorithms, and can achieve satisfactory results.

Figures 4, 5, 6, 7, 8, 9, 10, 11, 12, 13, 14, 15, 16 and 17 show the distributions of the PF and the real PF obtained by different algorithms in the ZDT6 and DTLZ5 benchmark functions. In Figs. 4, 5, 6, 7, 8, 9 and 10, the PF obtained by the WOF algorithm, the DGEA algorithm, the MOAHA algorithm and the NSCSO algorithm can cover the true PF without outliers with too large a gap, while the other algorithms can also achieve coverage of the true PF, but with too many outliers, uneven distribution and not wide coverage. In the DTLZ5 test problems in Figs. 11, 12, 13, 14, 15, 16 and 17, the PF obtained by the LMEA, the MOAHA and the NSCSO algorithms can achieve complete coverage of the true PF, but the MOAHA algorithm still has a small portion that is not covered, while there are also some Pareto solutions that have a certain distance from the true values. Figures 10 and 17 show the relationship plots of NSCSO. The PF obtained by NSCSO can cover the true Pareto solution completely and with uniform distribution. These images strengthen the support for the data in Tables 3, 4, 5 and 6. The PF obtained by the NSCSO algorithm are more uniform and have wider coverage. The superior performance of the NSCSO algorithm is further proved by the method of visualization.

**Figure 28:**
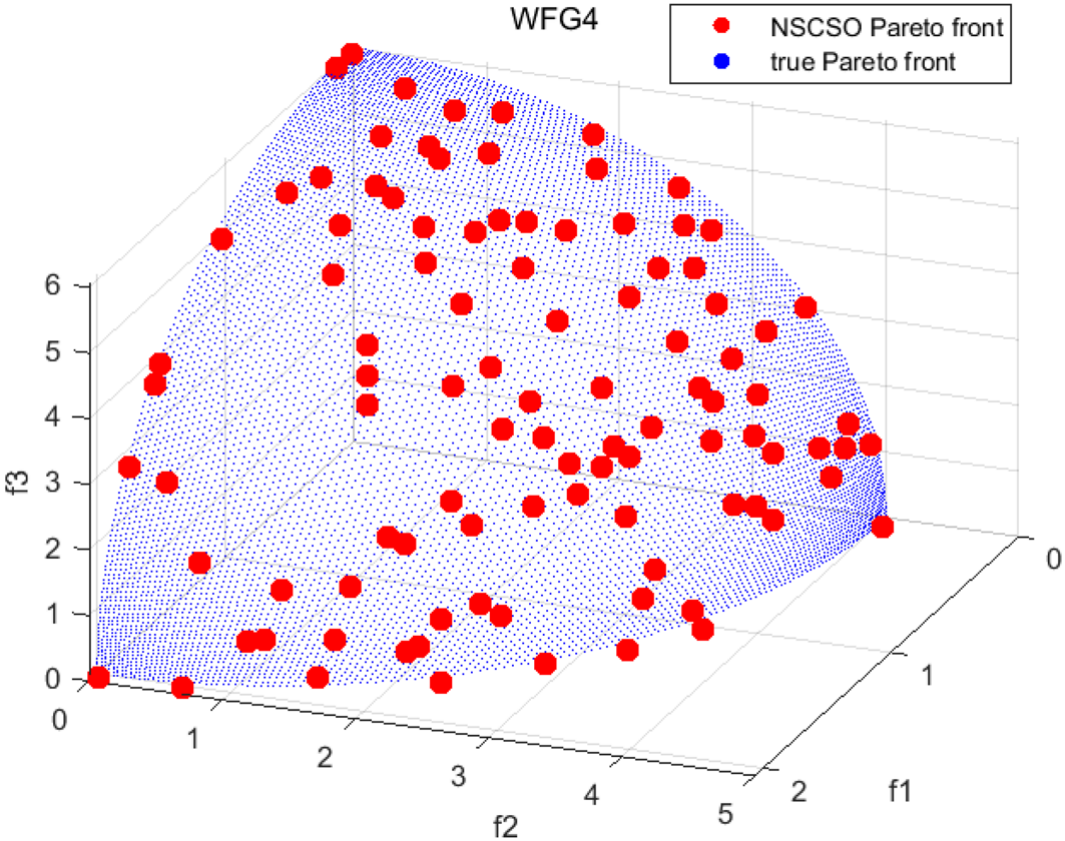
The Pareto front obtained by NSCSO on WFG4.

**Figure 29:**
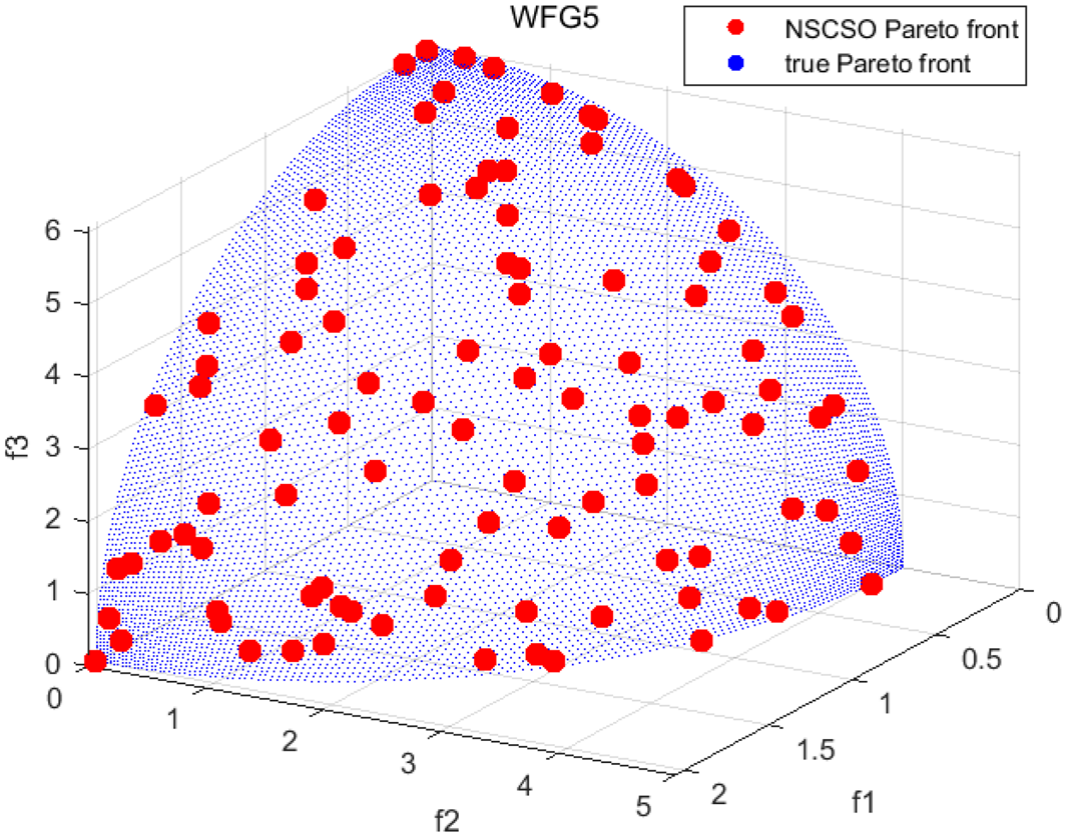
The Pareto front obtained by NSCSO on WFG5.

**Figure 30:**
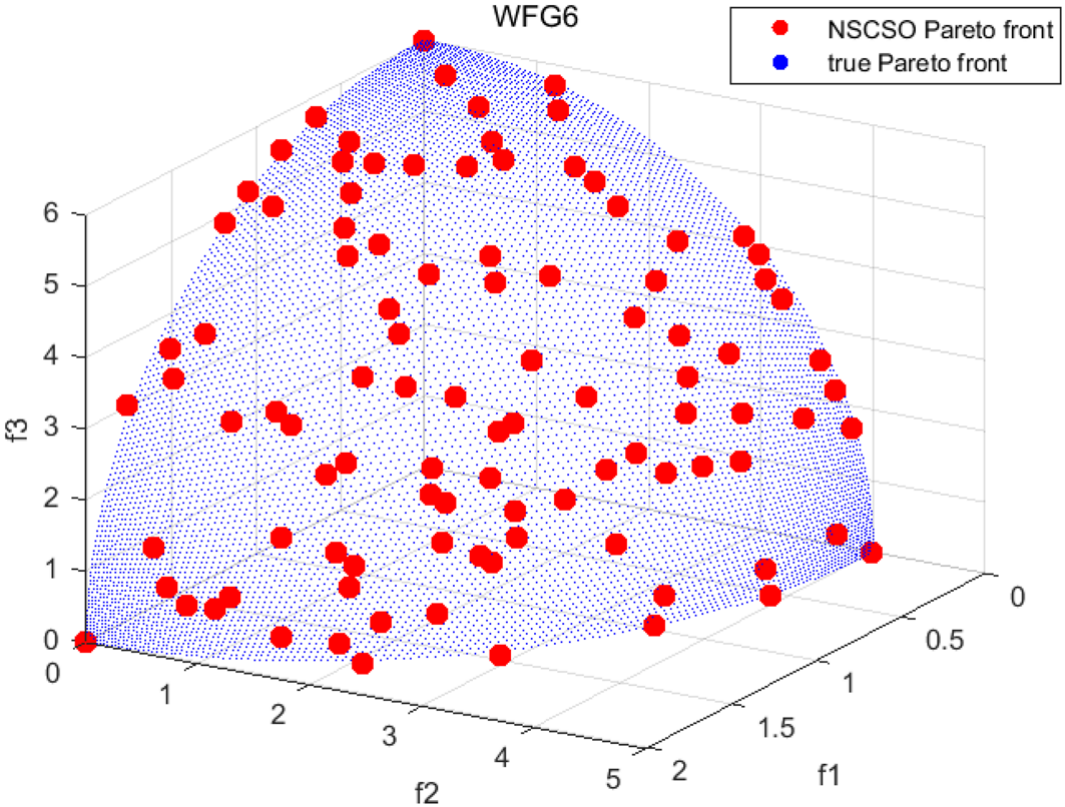
The Pareto front obtained by NSCSO on WFG6.

**Figure 31:**
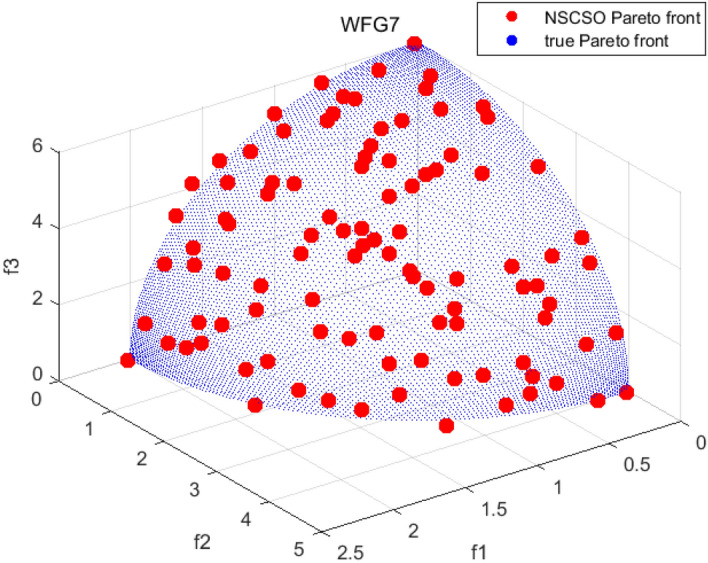
The Pareto front obtained by NSCSO on WFG7.

**Figure 32:**
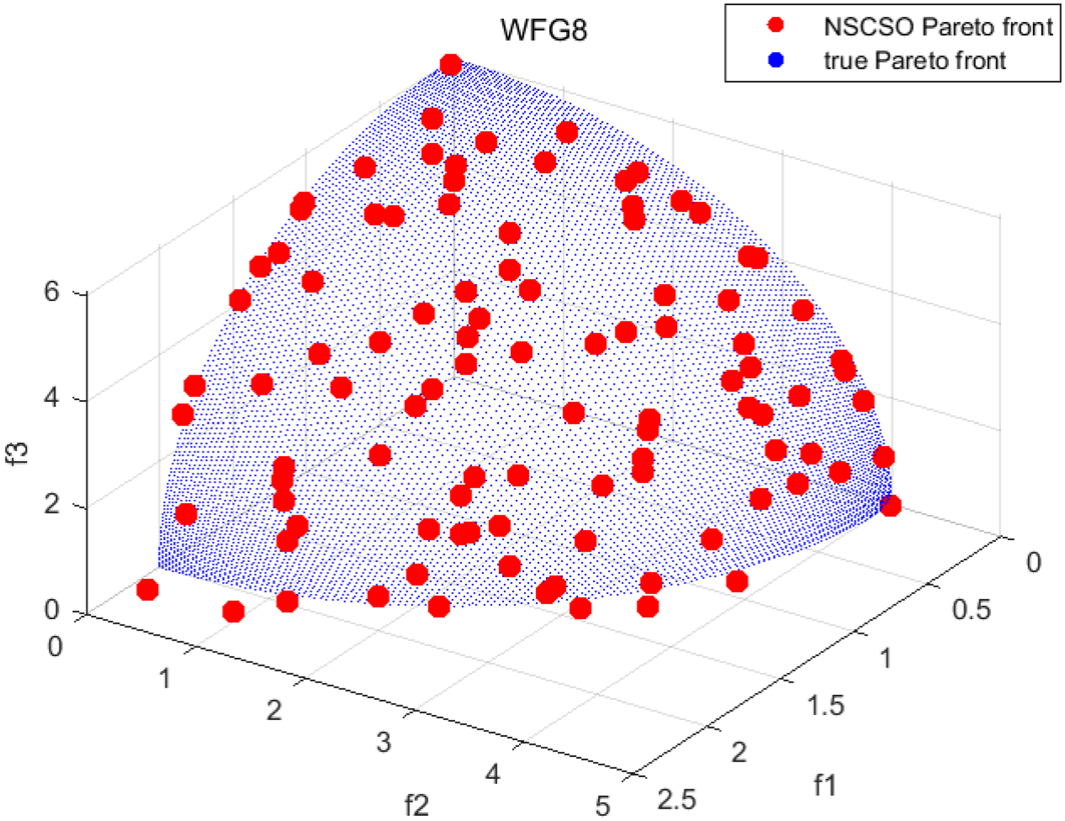
The Pareto front obtained by NSCSO on WFG8.

Figures 18, 19, 20, 21, 22, 23, 24, 25, 26, 27, 28, 29, 30, 31 and 32 show the plots of the PF obtained by the NSCSO algorithm for each benchmark function in the ZDT, DTLZ, and WFG test sets, respectively. By observing these figures, it can be found that in different test sets, the PF obtained by the NSCSO algorithm can be evenly distributed in the target space, the gap with the true value is also small, and the coverage of the true value is uniform, covering wide. Therefore, the NSCSO algorithm can obtain Pareto optimal solutions with excellent performance in different experimental environments.

Through the above analysis, the NSCSO algorithm performs well in these tests, and its superior global search capability enables to obtain the PF with the widest coverage and uniform distribution in all tests, and the algorithm has good convergence. Through the above analysis, it can be proved that the NSCSO algorithm can solve the MOP problem well.

### Friedman test

Friedman test to detect differences between algorithms. The NSCSO algorithm is made to compare with LMEA, WOF, MOSMA, DGEA, MOAHA, and MOSPO respectively. After passing the Friedman test, the p-value is output, which represents the impact of the target algorithm on NSCSO. If p < 0.05, it means that the two algorithms are different, otherwise it means that the two algorithms have some similarities.

The contents in Table 7 are the p-values obtained by the NSCSO algorithm compared with other algorithms through the comprehensive results of 15 test functions. After the Friedman test between the NSCSO algorithm and other algorithms, the obtained p-values are all less than 0.05, so the NSCSO algorithm is quite different from these algorithms. Therefore, the development of the NSCSO algorithm can provide a new way of thinking for our problem-solving. By comprehensive comparison of the test function results and Friedman test results, the results obtained by using the NSCSO algorithm to solve the MOP problem have better performance.

**Table 7:** Friedman test p-value.

	NSCSO vs LMEA	NSCSO vs WOF	NSCSO vs MOSMA	NSCSO vs DGEA	NSCSO vs MOAHA	NSCSO vs MOSPO
GD	1.075 × 10^–04^	1.075 × 10^–04^	1.075 × 10^–04^	1.075 × 10^–04^	1.075 × 10^–04^	1.075 × 10^–04^
IGD	1.075 × 10^–04^	1.075 × 10^–04^	1.075 × 10^–04^	1.075 × 10^–04^	4.509 × 10^–03^	1.075 × 10^–04^
SP	1.075 × 10^–04^	1.075 × 10^–04^	1.075 × 10^–04^	1.075 × 10^–04^	1.075 × 10^–04^	1.075 × 10^–04^
MS	1.075 × 10^–04^	1.075 × 10^–04^	1.075 × 10^–04^	1.075 × 10^–04^	7.891 × 10^–04^	1.075 × 10^–04^

## Engineering design problems

To further prove the effectiveness and feasibility of the NSCSO algorithm in practical applications, this paper compares the NSCSO algorithm with other multi-objective optimization algorithms in six different engineering design problems. Each algorithm is run 10 times in different engineering instances with 1000 iterations per iteration, the population size is 1000.

### Car side impact problem

**Figure 33:**
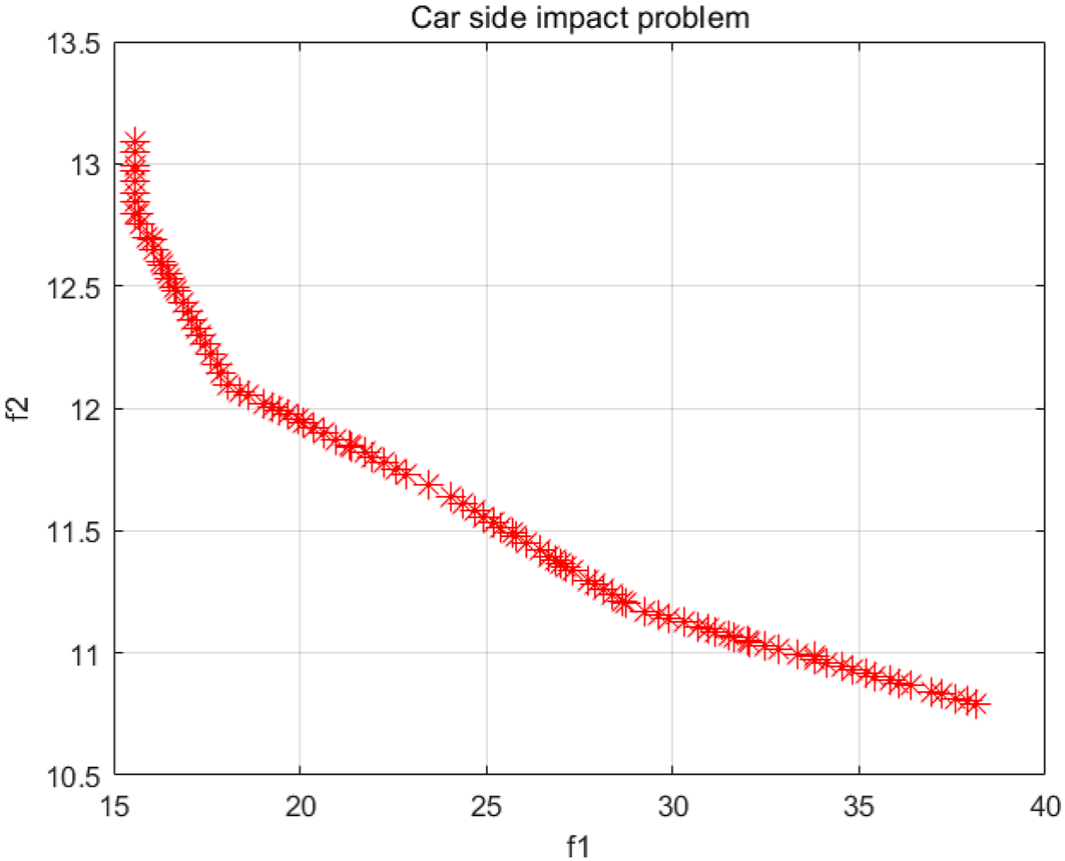
Pareto front obtained by NSCSO.

The car side impact problem generally involves seven variables^55^, parcel cross member, B-pillar inner panel thickness, door beltline reinforcement, B-pillar inner panel reinforcement, door beam, roof longitudinal beam and floor side inner panel. This problem is mainly used to optimize the constraint problem for the side impact resistance of the vehicle. The specific mathematical model is shown in Supplementary Appendix A. Table 8 shows the comparison of the SP values for this model, and Fig. 33 shows the Pareto front (PF) obtained by the NSCSO algorithm.

**Table 8:** Comparison of the SP solutions for the car side impact problem.

Algorithm	SP
MOSMA	0.1648
MOSPO	0.1156
NSCSO	**0.1153**

Significant values are in bold.

As shown in the data in Table 8, the NSCSO algorithm can obtain better PF than other algorithms. In Fig. 33, the PF of the NSCSO algorithm is evenly distributed with wide coverage. Therefore, it shows that the NSCSO algorithm shows its excellent performance in solving this problem.

### Gear train problem

**Figure 34:**
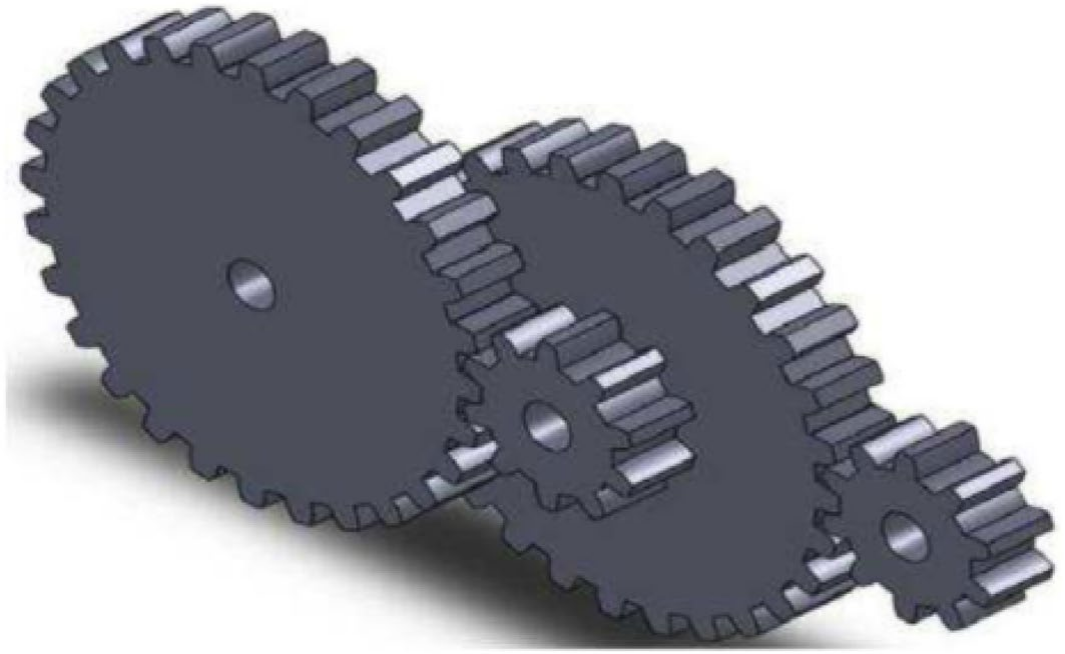
Gear train problem^57^.

**Figure 35:**
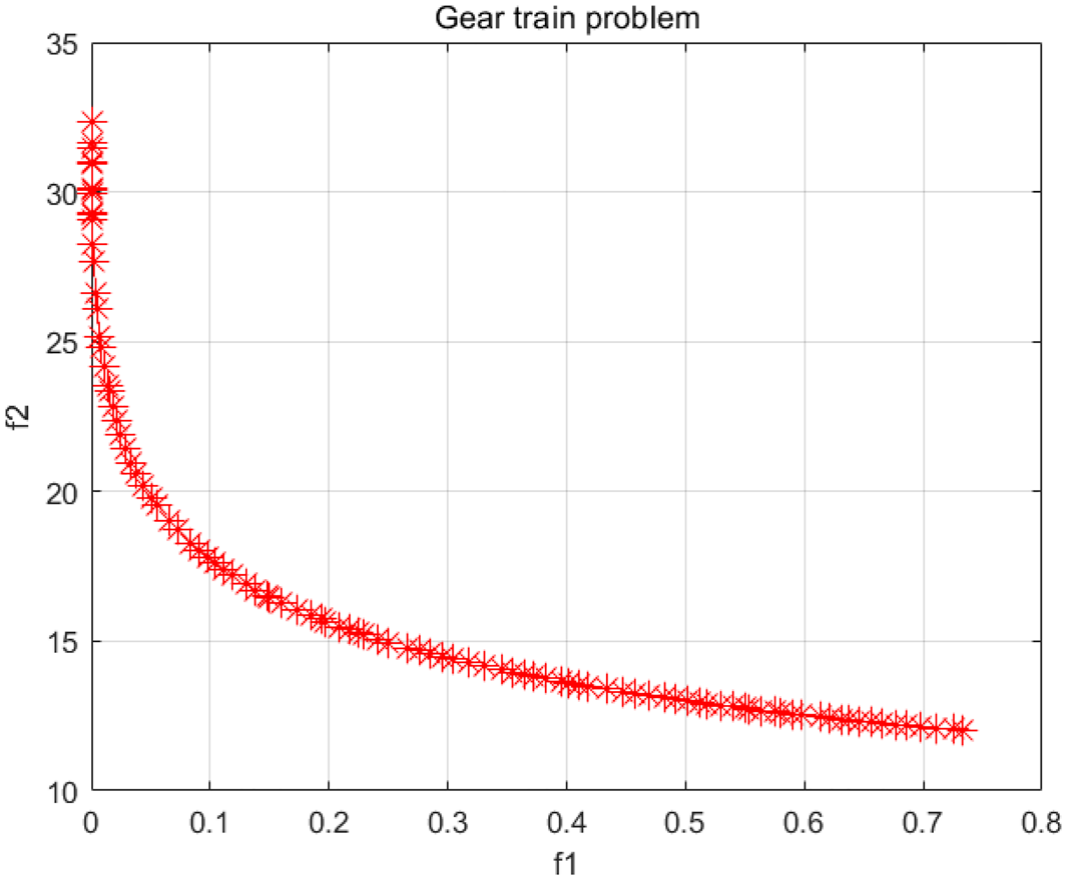
Pareto front obtained by NSCSO.

A gear train is a transmission system consisting of a set of gears. The goal of gear train design is to calculate the number of teeth for each gear in the gear train^56^, as shown in Fig. 34. The problem has four decision variables and two objective functions. At the same time, the decision variables of this problem are all integers, which are designed to follow realistic rules. Table 9 shows the SP values and extreme solutions obtained by different algorithms. Figure 35 shows the Pareto fronts obtained by the NSCSO algorithm. Supplementary Appendix B is the mathematical model.

**Table 9:** Comparison of the SP and extreme solutions for the gear train problem.

Algorithm	SP	Objective function	
		$f_{1}$	$f_{2}$
MOSMA	0.2905	–	–
$f_{1} \to \min$	–	6.00 × 10^–08^	39
$f_{2} \to \min$	–	7.32 × 10^–01^	12
MOSPO	0.1573		
$f_{1} \to \min$	–	2.65 × 10^–09^	40
$f_{2} \to \min$	–	7.32 × 10^–01^	12
**NSCSO**	**0.1126**	–	–
$f_{1} \to \min$	–	8.70 × 10^–11^	32
$f_{2} \to \min$	–	7.32 × 10^–01^	12

Significant values are in bold.

Through the data in Table 9, the NSCSO algorithm can obtain a smaller SP value than other algorithms, indicating that the distribution of its Pareto solution is the most uniform. Figure 35 also confirms this well. At the same time, it can be seen that the optimal extreme Pareto solution in the case of $f_{1} \to \min$ is computed by the NSCSO algorithm. All algorithms can obtain the ideal solution when $f_{2} \to \min$. Therefore, in this problem, the NSCSO algorithm can provide a better solution and provide a new choice for solving engineering problems.

### Welded beam design problem

**Figure 36:**
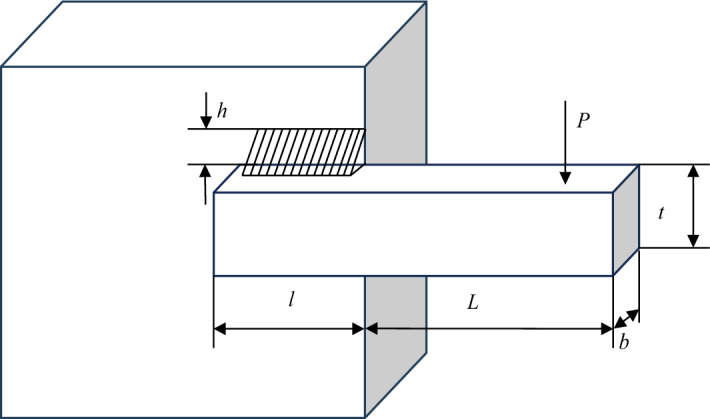
Welded beam design problem^58^.

**Figure 37:**
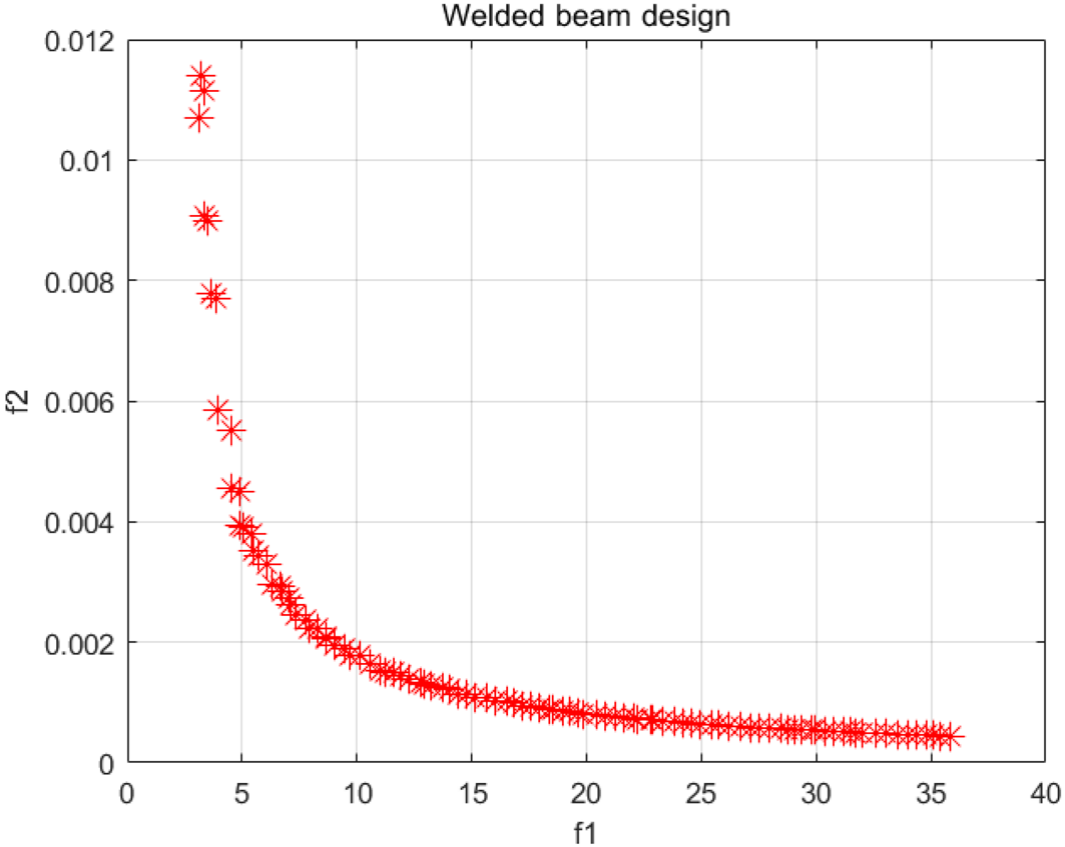
Pareto front obtained by NSCSO.

Welded beam design problem pursues the lowest production cost^58^. Its optimization goals include welding two vertical deflections and including manufacturing cost. As shown in Fig. 36, the decision variables for this problem are clip length $l + L$; rebar thickness $b$; rebar height $t$; weld thickness $h$; $P$ is the vertical deflection. Figure 37 is the PF graph obtained by the NSCSO algorithm for this problem. Table 10 shows the SP values for all algorithms. Supplementary Appendix C is the mathematical model.

**Table 10:** Comparison of the SP solutions for the welded beam design problem.

Algorithm	SP
MOSMA	0.6363
MOSPO	0.2971
NSCSO	**0.1348**

Significant values are in bold.

In this problem, the result obtained by the NSCSO algorithm ranks first, and the SP value is much smaller than that of MOSMA, and slightly smaller than that of MOSPO. The numerical gap also shows that the robustness of the NSCSO algorithm is far superior to several other algorithms. It can be seen from Fig. 37 that the PF distribution of the NSCSO algorithm is relatively uniform. Therefore, the NSCSO algorithm can provide more valuable reference data to help solve engineering problems through its superior performance and strong searchability.

### Cantilever beam design problem

**Figure 38:**
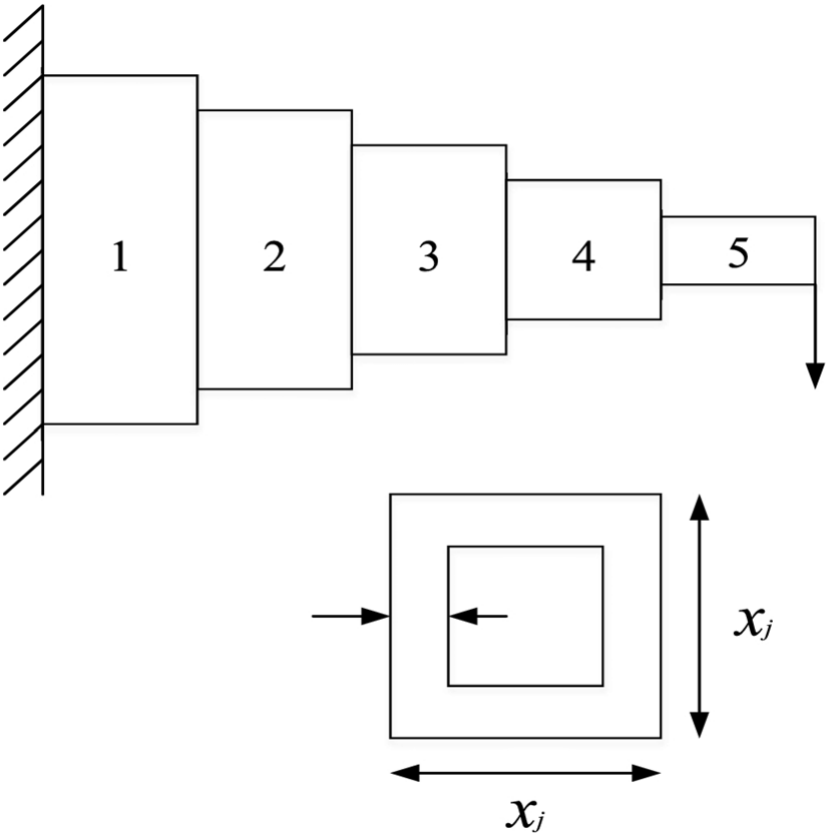
Cantilever beam design problem.

**Figure 39:**
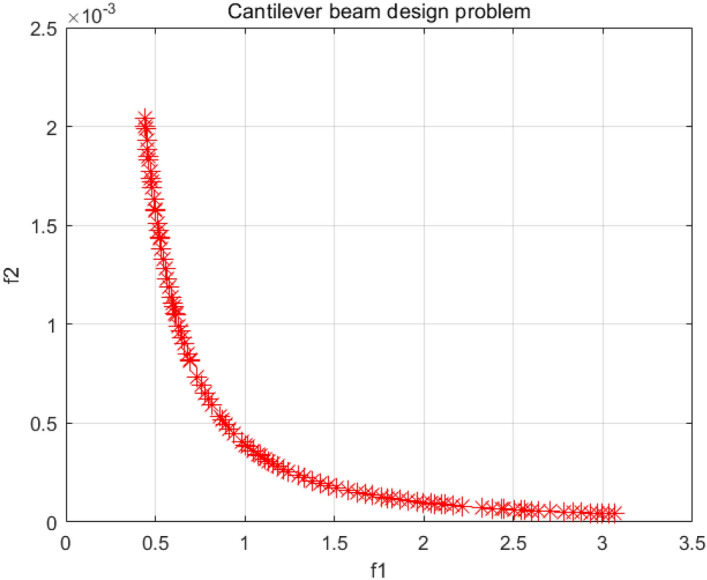
Pareto front obtained by NSCSO.

The cantilever beam design has two objective functions with the aim of optimizing its weight reduction and reducing the deflection of the cantilever beam under the constraints of maximum stress and maximum deflection^59^. As shown in Fig. 38, the problem is considered with one of its ends fixed and the diameter and length of its cross-section as decision variables. Figure 39 is the PF obtained by the NSCSO algorithm in this problem. The SP values of different algorithms for this problem are given in Table 11. Supplementary Appendix D shows the mathematical model.

**Table 11:** Comparison of the SP solutions for the cantilever beam design problem.

Algorithm	SP
MOSMA	0.0194
MOSPO	0.0964
NSCSO	**0.0124**

Significant values are in bold.

The distribution of the Pareto fronts obtained by the NSCSO algorithm in Fig. 39 is very uniform. Also, the data in Table 11 shows that the SP value of the NSCSO algorithm is the smallest among all algorithms. It is further proved that the solution obtained by the NSCSO algorithm is well distributed and has a good reference value.

### Disk brake design problem

**Figure 40:**
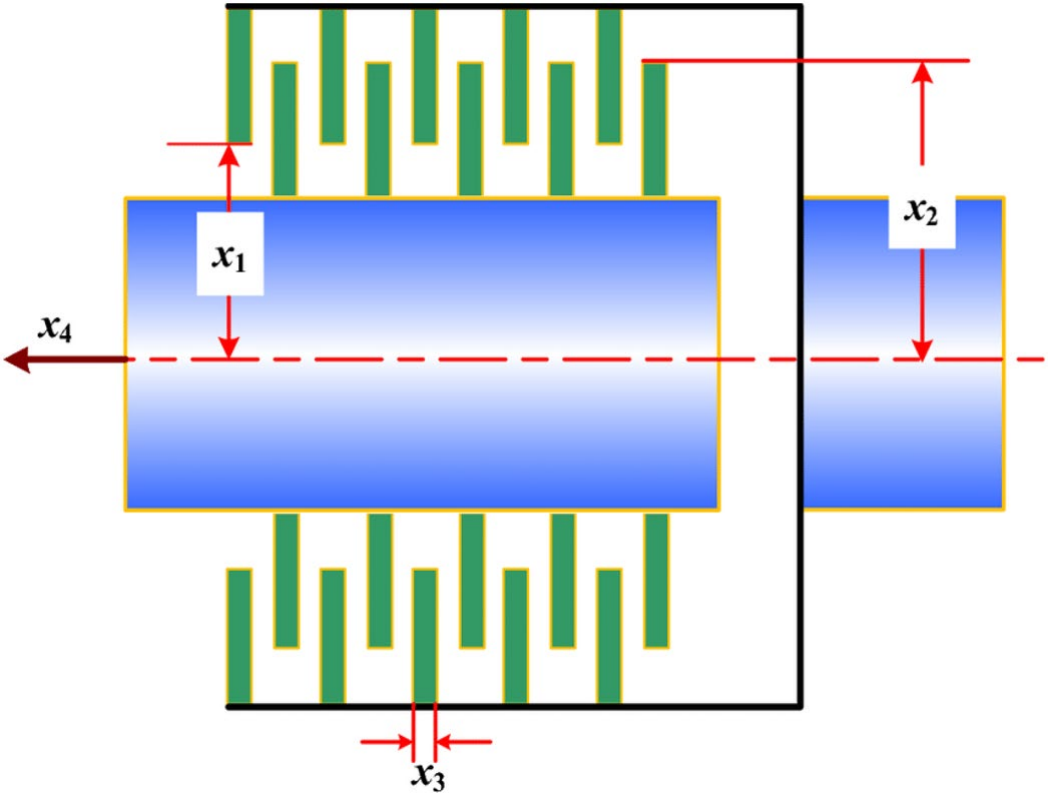
Disk brake design problem.

**Figure 41:**
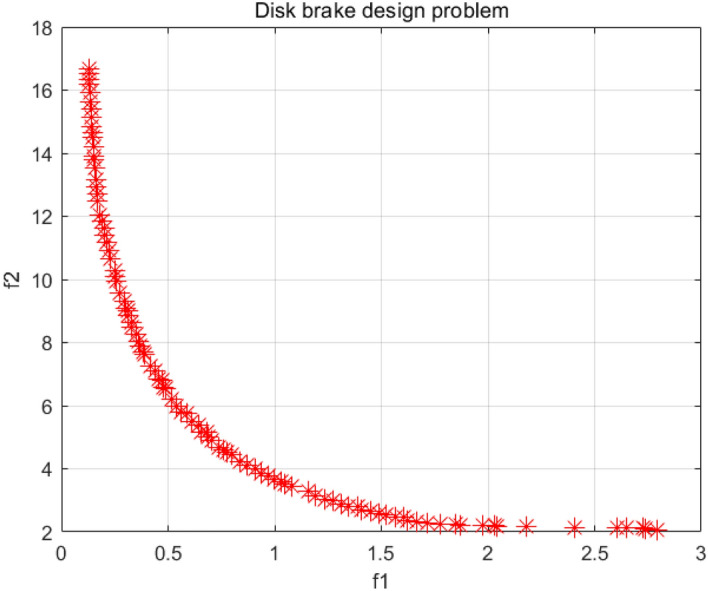
Pareto front obtained by NSCSO.

The two optimization objectives of the disc brake design problem are the stopping time and the quality of the braking system^60^. Figure 40 shows the model of this problem with four decision variables, which are the engagement force, the inner and outer radii of the table disc, and the number of friction surfaces. Figure 41 is the PF obtained by the NSCSO algorithm for this problem. Table 12 records the SP values and the extreme Pareto fronts for each algorithm, respectively. Supplementary Appendix E shows the mathematical model.

**Table 12:** Comparison of the SP and extreme solutions for the disk brake design problem.

Algorithm	SP	Objective function	
		$f_{1}$	$f_{2}$
MOSMA	0.2427	–	–
$f_{1} \to \min$	–	0.1274	16.6549
$f_{2} \to \min$	–	2.3581	2.4125
**NSCSO**	**0.0725**	–	–
$f_{1} \to \min$	–	0.1263	16.6549
$f_{2} \to \min$	–	2.7902	2.0729

Significant values are in bold.

In Table 12, the SP obtained by the NSCSO algorithm is much smaller than the SP obtained by MOSMA. The extreme values of the smallest $f_{1}$, $f_{2}$ can be obtained, and the extreme solutions of other algorithms are larger than those of the NSCSO algorithm. In Fig. 41, the NSCSO algorithm, although unevenly distributed around the range of 2–2.8, is already the best and most evenly distributed among all algorithms according to the comparison of SP values. Therefore, the PF obtained by the NSCSO algorithm is able to have good scalability, and in a realistic situation, it will be able to provide more excellent solutions for decision makers.

### Compression spring design problem

**Figure 42:**
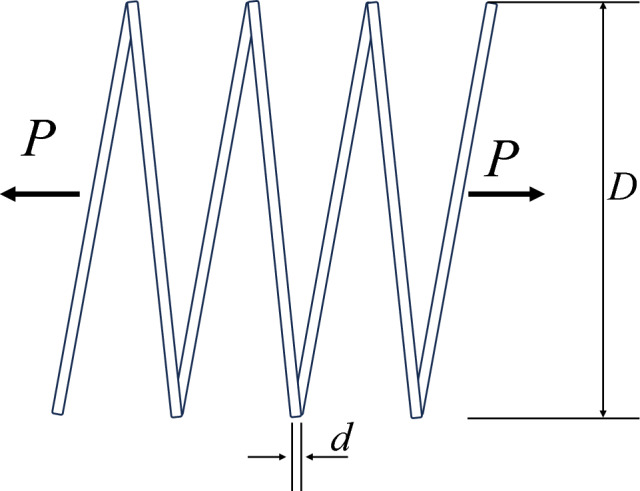
Compression spring design problem.

**Figure 43:**
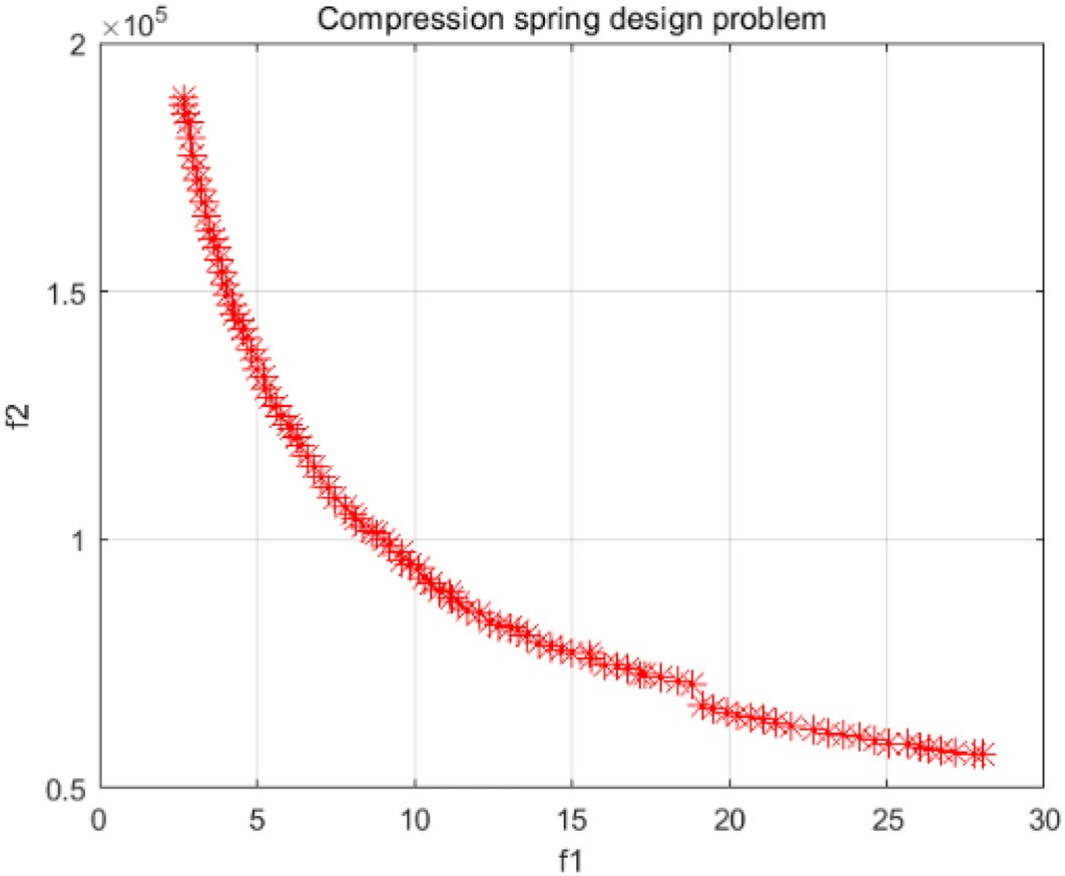
Pareto front obtained by NSCSO.

The pressure spring design problem is a discrete problem with the objective of reducing its pressure and volume^61^. Figure 42 shows the model for this problem with four decision variables, the average coil diameter (D), the wire diameter (d), and the number of active coils (P). Figure 43 is the PF plot obtained for the optimization of this problem using the NSCSO algorithm. Table 13 shows the extreme PF solutions obtained by different algorithms. Supplementary Appendix F for the mathematical model.

**Table 13:** Comparison of the extreme solutions for the compression spring design problem.

Algorithm	Objective function	
	$f_{1}$	$f_{2}$
MOAHA		
$f_{1} \to \min$	2.68	187,966
$f_{2} \to \min$	27.99	56,748
MOSPO		
$f_{1} \to \min$	2.78	187,191
$f_{2} \to \min$	27.82	57,104
NSCSO		
$f_{1} \to \min$	2.65	188,987
$f_{2} \to \min$	28.01	56,697

As can be seen in Table 13, NSCSO is able to obtain extreme solutions that cannot be obtained by other algorithms when the constraints are satisfied. Also, the distribution of the PF of the NSCSO algorithm in Fig. 43 is very uniform. Since the line diameter in this problem is a discrete value and there is a certain Pareto front for different discrete values, it can be seen that there are discontinuities or overlaps in Fig. 43. This also indicates the correctness of the results.

By testing the above six engineering examples, the PF obtained by the NSCSO algorithm in these cases is more evenly distributed and has better performance than other algorithms. Therefore, this also proves that NSCSO not only performs superiorly in multi-objective function tests but also obtains realistic solutions in multi-objective engineering instance problems, providing new options for solving engineering instance problems and broadening the usability of the NSCSO algorithm.

## Conclusions

In this study, a novel multi-objective variant of the chicken swarm optimization (CSO) algorithm is proposed, referred to as non-dominated sorting chicken swarm optimization (NSCSO). The algorithm employs fast non-dominated sorting and crowding distance strategies to rank individuals based on their fitness, allocating roles while preserving the original CSO algorithm’s hierarchical structure and obtaining Pareto optimal solutions. Through an elite reverse learning mechanism, individual chickens are guided towards exploring the optimal solution direction, facilitating knowledge transfer to other particles and enhancing the algorithm’s search capability. The algorithm is extensively tested on various benchmark datasets and subjected to Friedman tests. Ultimately, when compared to LMEA, WOF, MOSMA, DGEA, MOAHA, and MOSPO, the NSCSO algorithm consistently yields superior Pareto optimal solution sets. Furthermore, the NSCSO algorithm is applied to address six practical engineering problems, demonstrating its effectiveness in solving real-world issues and expanding the algorithm’s applicability.

Future work will involve further refinement of the NSCSO algorithm and its application to more intricate practical scenarios, such as microgrid allocation problems^62^, WSN node coverage problems^63^. Additionally, the proposed algorithm’s multi-objective version holds promise as a valuable contribution for future research endeavors.

### Supplementary Information

Supplementary Information.

## Data Availability

All data generated or analyzed during this study are included in this published article.
