# Supplementary material for: NSCSO: a novel multi-objective non-dominated sorting chicken swarm optimization algorithm
Source: Sci Rep. 2024 Feb 21;14:4310. doi: 10.1038/s41598-024-54991-0 (PMC10881516; doi:10.1038/s41598-024-54991-0)
Supplement: Supplementary file 1 — Supplementary Information. [file 41598_2024_54991_MOESM1_ESM.docx]

Appendix: Engineering Design Problems

1. Car Side Impact Problem [54]

Objective function:

$f_{1}(x)=1.98\text{+}4.9x_{1}\text{+}6.67x_{2}\text{+}6.98x_{3}\text{+}4.01x_{4}\text{+}1.78x_{5}\text{+}0.00001x_{6}\text{+}2.73x_{7}$ $f_{2}(x)=0.5*(10.58\text{-}0.674x_{1}x_{2}\text{-}0.67275x_{2}\text{+}16.45\text{-}0.489x_{3}x_{7}\text{-}0.843x_{5}x_{6})$

Subject to:

$$g_{1}(x)\text{ }=\text{ }1.16-0.3717x_{2}x_{4}-0.0092928x_{3}\leq1.0$$

$$g_{2}(x)\text{ }=\text{ }0.261-0.0159x_{1}x_{2}-0.06486x_{1}-0.019x_{2}x_{7}+0.0144x_{3}x_{5}+0.0154464x_{6}\leq0.32$$

$$\begin{matrix} g_{3}(x)\text{ }=\text{ }0.214+0.00817x_{5}-0.045195x_{1}-0.0135168x_{1}+0.03099x_{2}x_{6}-0.018x_{2}x_{7}+0.007176x_{3}+0.023232x_{3}- \\ \begin{matrix} & \begin{matrix} & \begin{matrix} & 0.00364x_{5}x_{6}-0.018x_{2}x_{2}\leq0.32 \end{matrix} \end{matrix} \end{matrix} \end{matrix}$$

$$g_{4}(x)\text{ }=\text{ }0.74-0.61x_{2}-0.031296x_{3}-0.031872x_{7}\text{+}0.227x_{2}x_{2}\leq0.32$$

$$g_{5}(x)\text{ }=\text{ }28.98+3.818x_{3}-4.2x_{1}x_{2}+1.27296x_{6}-2.68065x_{7}\leq0.32$$

$$g_{6}(x)=33.86+2.95x_{3}-5.057x_{1}x_{2}-3.795x_{2}-3.4431x_{7}+1.45728\leq0.32$$

$$g_{7}(x)=46.36-9.9x_{2}-4.4505x_{1}\leq0.32$$

$$g_{8}(x)=4.72-0.5x_{4}-0.19x_{2}x_{3}\leq4.0$$

$$g_{9}(x)=10.58-0.674x_{1}x_{2}-0.67275x_{2}\leq9.9$$

$$g_{10}(x)=16.45-0.489x_{3}x_{7}-0.843x_{5}x_{6}\leq15.7$$

where,

$$\begin{matrix} 0.5\leq x_{1},x_{3},x_{4}\leq1.5, \\ 0.4\leq x_{6},x_{7}\leq1.2, \\ 0.45\leq x_{2}\leq1.35, \\ 0.875\leq x_{5}\leq2.625. \end{matrix}$$

1. Gear Train Problem [55]

Minimize: $\text{ }f_{1}(x)=((\frac{1}{6.931})-(\frac{x_{1}x_{2}}{x_{3}x_{4}}))^{2}$

Minimize:$\text{ }f_{2}(x)=\max(x_{1},x_{2},x_{3},x_{4})$

where, $12\leq x_{1},x_{2},x_{3},x_{4}\leq60$

1. Welded Beam Design Problem [57]

Minimize:$\text{ }f_{1}(x)=1.10471x_{1}^{2}x_{2}+0.04811(14.0+x_{2})x_{3}x_{4}$

Minimize:$\text{ }f_{2}(x)=65856000/(30*10^{6}x_{4}x_{3}^{3})$

where,

$$\begin{matrix} g_{1}(x)=\tau-13600 \\ g_{2}(x)=\sigma-13600 \\ g_{3}(x)=x_{1}-x_{4} \\ g_{4}(x)=6000-P \end{matrix}$$

$$\begin{matrix} 0.125\leq x_{1}\leq5, \\ 0.1\leq x_{2}\leq10, \\ 0.1\leq x_{3}\leq10, \\ 0.125\leq x_{4}\leq5. \end{matrix}$$

where,

$$\begin{matrix} q=6000*(14+\frac{x_{2}}{2})\text{，} \\ D=sqrt(\frac{x_{2}^{2}}{4}+\frac{(x_{1}-x_{3})^{2}}{4})\text{，} \end{matrix}$$

$$J=2*(x_{1}x_{2}*\sqrt{2}*(\frac{x_{2}^{2}}{12}+\frac{(x_{1}+x_{3})^{2}}{4}))\text{，}$$

$$\begin{matrix} \alpha=\frac{6000}{sqrt(2)*x_{1}*x_{2}}\text{，} \\ \beta=Q*\frac{D}{J}\text{，} \\ \tau=sqrt(\alpha^{2}+2\alpha\beta*\frac{x_{2}}{2D}+\beta^{2})\text{，} \end{matrix}$$

$$\begin{matrix} \sigma=\frac{504000}{x_{4}x_{3}^{2}}\text{，}tmpf=4.013*\frac{30*10^{6}}{196}\text{，} & & \end{matrix}$$

$$P=tmpf*sqrt(x_{3}^{2}\frac{x_{4}^{6}}{36})*(1-x_{3}\frac{sqrt\frac{30}{48}}{28})$$

1. Cantilever Beam Design Problem [58]

Minimize:$f_{1}(x)=0.25\rho\pi x_{2}x_{1}^{2}$

Minimize:$f_{2}(x)=(64Px_{2}^{3})/(3E\pi x_{1}^{4})$

where,

$$\begin{matrix} g_{1}(x)=-Sy+(32Px_{2})/(\pi x_{1}^{3}) \\ g_{2}(x)=-\delta_{\max}+(64Px_{2}^{3})/(3E\pi x_{1}^{4}) \end{matrix}$$

$$\begin{matrix} 0.01\leq x_{1}\leq0.05 \\ 0.20\leq x_{2}\leq1 \end{matrix}$$

where

$$\begin{matrix} P=1, \\ E=207000000, \\ S_{y}=300000, \\ \delta_{\max}=0.005, \\ \rho=7800. \end{matrix}$$

1. Disk Brake Design Problem [59]

Minimize:$f_{1}(x)=(4.9e-5)(x_{2}^{2}-x_{1}^{2})(x_{4}-1)$

Minimize:$f_{2}(x)=(9.82e6)\frac{x_{2}^{2}-x_{1}^{2}}{x_{3}x_{4}(x_{2}^{3}-x_{1}^{3})}$

$$\begin{matrix} g_{1}(x)=20+x_{1}-x_{2} \\ g_{2}(x)=2.5(x_{4}+1)-30 \end{matrix}$$

$$\begin{matrix} g_{3}(x)=\frac{x_{3}}{\pi(x_{2}^{2}-x_{1}^{2})^{2}}-0.4 \\ g_{4}(x)=(2.22e-3)x_{3}\frac{x_{2}^{3}-x_{1}^{3}}{(x_{2}^{2}-x_{1}^{2})^{2}}-1 \end{matrix}$$

$$g_{5}(x)=900-(\frac{(2.66e-2)x_{3}x_{4}(x_{3}^{3}-x_{1}^{3})}{x_{2}^{2}-x_{1}^{2}})$$

where,

$$\begin{matrix} \forall g\leq0, \\ 55\leq x_{1}\leq80, \\ 75\leq x_{2}\leq110, \\ 1000\leq x_{3}\leq3000, \\ 2\leq x_{4}\leq20, \\ x_{4}\in I. \end{matrix}$$

1. Compression Spring Design Problem [60]

Minimize: $\text{ }f_{1}(x)=(0.25pi^{2}x_{2}^{2}x_{3}(x_{1}+2))$

Minimize: $\text{ }f_{2}(x)=8KP_{\max}\frac{x_{3}}{pix_{2}^{3}}$

where,

$$g_{1}(x)=1.05x_{2}(x_{1}+2)+\frac{P_{\max}}{k}-l_{\max}$$

$$g_{2}(x)=d_{\max}-x_{2}$$

$$g_{3}(x)=x_{2}+x_{3}-D_{\max}$$

$$g_{4}(x)=3-C$$

$$g_{5}(x)=del_{p}-del_{pm}$$

$$g_{6}(x)=del_{w}-\frac{P_{\max}-P}{k}$$

$$g_{7}(x)=8KP_{\max}\frac{x_{3}}{pix_{2}^{3}}-S$$

$$g_{6}(x)=(0.25pi^{2}x_{2}^{2}x_{3}(x_{1}+2))-V_{\max}$$

where,

$$\begin{matrix} P=300, \\ D_{\max}=3, \\ V_{\max}=30, \\ P_{\max}=1000, \\ del_{w}=1.25, \\ l_{\max}=14, \end{matrix}$$

$$\begin{matrix} d_{\min}=0.2, \\ S=189000, \\ G=11500000, \\ C=\frac{x_{3}}{x_{2}}, \\ k=G\frac{x_{2}^{4}}{8x_{1}x_{3}^{3}}, \end{matrix}$$

$$del_{p}=\frac{P}{k},K=\frac{4C-1}{4C-4}+(0.65\frac{x_{2}}{x_{3}}),$$

$$\begin{matrix} \forall g\leq0, \\ 1\leq x_{1}\leq32, \\ 1\leq x_{3}\leq30, \\ x_{1}\in I \end{matrix}$$

$$\begin{matrix} x_{2}\in[0.009,0.0095,0.0104,0.0118,0.0128,0.0132,0.014,0.014,0.0162,0.0173,0.018,0.020,0.023,0.025,0.028,0.032, \\ \text{ }0.035,0.041,0.047,0.054,0.063,0.072,0.192,0.207,0.225,0.244,0.263,0.283,0.307,0.331,0.362,0.394,0.4375,0.5] \end{matrix}$$
